# Synthesis, Characterization, and Crystal Structures of Imides Condensed with *p*-Phenylamino(Phenyl) Amine and Fluorescence Property

**DOI:** 10.3390/ma12111873

**Published:** 2019-06-10

**Authors:** Jing Zhang, Huaibo Ma

**Affiliations:** Key Laboratory of Flexible Electronics (KLOFE), Institute of Advanced Materials (IAM), Nanjing Tech University, 30 South Puzhu Road, Nanjing 211816, China; iamzj@njtech.edu.cn

**Keywords:** imide, *p*-phenylamino(phenyl)amine, X-ray crystal structure, hydrogen bond, fluorescence, electrochemistry

## Abstract

A series of aromatic diimide and monoimide compounds condensed with *p*-phenylamino(phenyl)amine were synthesized and confirmed by Proton Nuclear Magnetic Resonance (^1^H NMR), Carbon-13 Nuclear Magnetic Resonance (^13^C NMR), Fourier Transform Infrared Spectroscopy (FT-IR), Elemental Analysis (EA), and High Resolution Mass Spectroscopy (HRMS). Meanwhile, single crystal X-ray diffraction showed the existence of intermolecular N···O hydrogen bonds, which affected the thermal stabilities of corresponding compounds by the support of Thermalgravimetric Analysis (TGA) curves. The steady-state UV-vis absorption peaks of synthetic compounds **1**–**6** appeared in the range of 220–380 nm. Fluorescence emission spectra showed peaks in the range of 290–420 nm. Meanwhile, deep-blue or violet-blue emissions for **2**, **4**, and **5** in THF under excitations of 254 nm and 365 nm, respectively, were observed at room temperature in air. Furthermore, Differential pulse voltammetry (DPV) and cyclic voltammogram CV were conducted within −1.5–+1.5 V to show quasi-reversible behavior for conjugated compounds and irreversible behavior for less conjugated ones.

## 1. Introduction

Conjugated organic polymers and small molecules display both charge and energy transfer properties through π–π or π–n–π, which are affected by intrinsic structures, packing modes, and morphology [1,2,3]. Applications were found in organic nonvolatile memories (ONVMs) [4], organic field-effect transistors (OFETs) [5,6], single molecular switch [7], photonics [8], organic solar cells (OSCs) [9], organic rechargeable lithium-/sodium ion batteries [10,11,12,13], and electrochromism [14,15,16]. Particularly, rylene diimides were widely studied, due to their high electron mobilities because of long range conjugation and high thermal and oxidative stabilities [17]. Moreover, multifunctional properties on rylene diimides were also explored as molecular imaging agents [18,19] and probes [20,21,22]. A few rylene diimides, such as perylene diimide dyes [23,24] and naphthalene diimide derivatives [25,26,27], showing fluorescence property in solid state were synthesized with non-arylamine, regardless of the aggregation-caused quenching (ACQ) effect, leading to weak or quenched fluorescence property in solid state [28].

To investigate the binding process for small molecules to DNA motivated by discovering drugs, fluorescence property on both naphthalimide-based Schiff base derivatives, reported by Uddin [29], and 1,4,5,8-naphthalene diimide–spermine conjugate (NDIs), by Wang [30], was exploited. The latter case also revealed that hydrogen bonding interactions play an important role in fluorescence quenching. A water-soluble fluorescent probe of naphthalene diimide combined with red and blue NDIs was reported by Freccero [31], which gave a red/NIR emission upon binding with G-quadruplex. Furthermore, conjugated aromatic compounds containing oxadiazole and pyridine units upon coordination with zinc ions by Diana [32] showed bright luminescence. In addition, Caruso reported a fluorescence sensor, pyridyl/phenolic/benzothiazole functionalized colorimetric receptors (BPAP), with high selectivity and excellent sensitivity towards zinc and cadium ions [33].

N, N′-bis[*p*-phenylamino(phenyl)]-1,4,5,8-naphthalenetetracarboxylic diimide (DNTD) and N, N′-bis[*p*-phenylamino(phenyl)]-3,4,9,10-perylenetetracarboxylic diimide (DPTD) were reported by Cammarata [34,35,36] and Guo [37,38,39], respectively. Single crystal structures provide rich information about packing and weak interactions, including hydrogen bonds, π–π stacking, and the van der Waals force. Detailed structural data are not only useful to understand mechanisms but also important for guiding the design of new molecules. However, such conjugated imides have low solubility issues in many organic solvents.

**Scheme 1:**
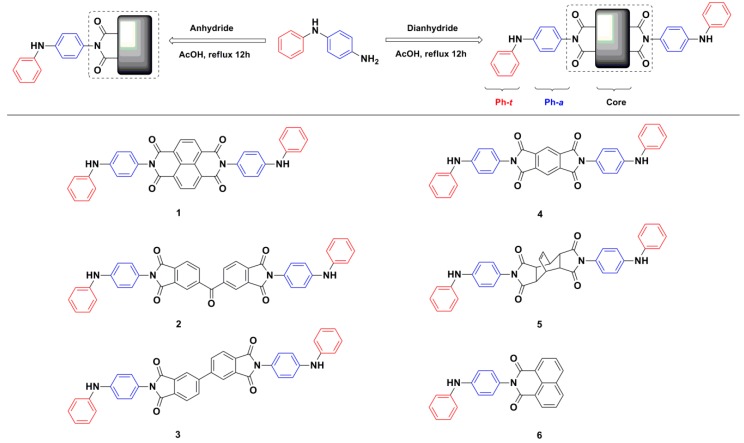
Synthesis of diimide and monoimide compounds.

In this work, we describe single crystal structures and thermal stability, fluorescence, and electrochemical properties based on synthesized imide compounds. Herein, a series of diimide compounds **1**–**5** and monoimide compound **6** condensed with *p*-phenylamino(phenyl)amine were successfully synthesized (Scheme 1). Of these, **2**–**6** are new compounds and single crystal structures of **1**, **3**, and **5** are firstly presented. Furthermore, the roles of intermolecular N···O hydrogen bonds on thermal stability and fluorescence properties are discussed accordingly.

## 2. Materials and Methods

All commercially available starting materials, reagents, and solvents were purchased and used as supplied. *p*-Phenylamino(phenyl)amine, Naphthalene-1,4,5,8-tetracarboxylic dianhydride, Pyromellitic dianhydride, and 4,4′-Biphthalic anhydride were purchased from TCI (Shanghai, China). Bicyclo[2.2.2]oct-7-ene-2,3,5,6-tetracarboxylic dianhydride, 1,8-Naphthalene anhydride, and 3,3′,4,4′-Benzophenonetetracarboxylic dianhydride were purchased from Alfa Aesar. Acetic acid was purchased from Greagent. Infrared spectra (KBr pellets) were measured on a Bruker MPA spectrometer (Billerica, MA, USA) between 400 and 4000 cm^−1^. Thermogravimetric analysis (TGA) was measured under a nitrogen stream, at a flow rate of 50 cm^3^/min with a heating rate of 10 °C/min on Mettler TGA2 Thermogravimetric Analyzer (Zurich, Switzerland). ^1^H NMR and ^13^C NMR spectra were recorded on either a Bruker ADVANCE AV-300 or ADVANCE-500 spectrometer (Billerica, MA, USA). Elemental analysis data were obtained using an instrument of Vario EL cube Elementar (Hamburg, Germany). Fluorescence spectra were obtained using a Hitachi F-4600 fluorescence spectrophotometer (Tokyo, Japan). Photoluminescent quantum yields (PLQYs) were obtained using a Fluoromax-4C-L TCSPC spectrophotometer (Horiba, Kyoto, Japan) at room temperature in air. UV-vis absorption spectra were obtained using a Shimadzu UV-1750 spectrometer (Kyoto, Japan). High resolution mass spectra (HRMS) were obtained using an Agilent 1260-6230 sepectrometer (Santa Clara, CA, USA). Differential pulse voltammetry (DPV) and cyclic voltammogram (CV) spectra were conducted with a three-electrode system on a Pine WaveDriver200 electrochemical workstation (Phoenix, AZ, USA), with a scanning rate of 0.1 Vs^−1^ in 0.1 M TBAPF_6_ DMF solutions. Pt wire and Pt disk were used as a counter electrode and a working electrode, respectively. Ag/AgCl was used as a reference electrode. Single crystal X-ray diffraction data were collected on a Bruker APEX-II diffractometer (Billerica, MA, USA) at 296 (2) K equipped with a CCD detector. The X-ray beam was generated using graphite monochromated Mo Kα radiation (λ = 0.71073 Å). All single crystal structures were solved by direct method with structure refinements being performed by the SHELXTL program [40]. Hydrogen atoms were introduced in calculated positions and refined according to the riding model. Figures related to crystal structures were generated by Mercury 3.10.2. The synthesis of compound **1** followed a modified procedure [41]. 

**N, N′-bis[*p*-phenylamino(phenyl)]-1,4,5,8-naphthalenetetracarboxylic diimide (DNTD) 1**

Naphthalene-1,4,5,8-tetracarboxylic dianhydride (1.34 g, 5 mmol) was directly added to 120 mL glacial acetic acid dissolved with *p*-phenylamino(phenyl)amine (2.76 g, 15 mmol) and then heated to reflux for 12 h. The solution was cooled down to room temperature and poured into methanol (250 mL), resulting in a precipitate. The crude product was collected by filtration then suspended in 250 mL methanol. Under sonication for five minutes, the purified solid was recollected by filtration. This operation was repeated three times to obtain a purple product (2.70 g, 4.50mmol, 90%). Purple crystals were obtained by evaporation of CH_3_CN/DMF (1:4) at room temperature. ^1^H NMR (500 MHz, DMSO-d_6_, ppm): 8.72 (s, 2H, Core*H*), 8.37 (s, 1H, N*H*), 7.25–7.31 (m, 4H, Ar*H*), 7.19 (t, 4H, Ar*H*), 6.89 (t, 1H, Ar*H*); ^13^C NMR (300 MHz, DMSO-d_6_/TFA-d, ppm): 140.79, 140.37, 138.83, 136.58, 135.32, 134.92, 134.81, 131.32, 130.77, 128.50, 126.32, 124.24, 120.48, 116.72, 112.97; FT-IR (KBr pellet, cm^−1^): 1666(s), 1592(s), 1581(m), 1532(s), 1509(m), 1496(s), 1448(m), 1409(w), 1345(m), 1328(s); HRMS(APCI) m/z calcd: 601.1876 [M+H]^+^, found 601.1851; Anal. calcd for C_38_H_24_N_4_O_4_ (%): C 75.99; H 4.03; N 9.33, found: C 74.55; H 4.03; N 9.14.

**N, N′-bis[*p*-phenylamino(phenyl)]-3,3′,4,4′-benzophenonetetracarboxylic diimide (DBTD) 2**

A similar method as described above was employed by the substitution of naphthalene-1,4,5,8-tetracarboxylic dianhydride with 3,3′,4,4′-benzophenonetetracarboxylic dianhydride (1.61 g, 5 mmol). A brick red product was obtained (3.05 g, 4.66 mmol, 93%). ^1^H NMR (500 MHz, DMSO-d_6_, ppm): 8.39 (s, 1H, N*H*), 8.25 (d, 1H, Core*H*), 8.16 (d, 2H, Core*H*), 7.28 (t, 4H, Ar*H*), 7.14–7.19 (m, 4H, Ar*H*), 6.89 (t, 1H, Ar*H*); ^13^C NMR (300 MHz, DMSO-d_6_/TFA-d, ppm): 199.38, 145.82, 140.70, 139.42, 138.69, 138.43, 136.85, 135.20, 134.78, 134.51, 134.40, 132.65, 129.28, 128.65, 127.59, 126.02, 124.00, 120.24, 116.48, 112.72; FT-IR (KBr pellet, cm^−1^): 1715(s), 1654(m), 1603(m), 1527(m), 1504(w), 1494(w), 1347(w), 1322(m); HRMS(APCI) m/z calcd: 655.1981 [M+H]^+^, found 655.1965; Anal. calcd for C_41_H_26_N_4_O_5_ (%): C 75.22; H 4.00; N 8.56, found: C 75.70; H 3.95; N 8.56.

**N, N′-bis[*p*-phenylamino(phenyl)]-4,4′-biphthalic diimide (DBD) 3**

A similar method as described above was employed by the substitution of naphthalene-1,4,5,8-tetracarboxylic dianhydride with 4,4′-biphthalic anhydride (1.47 g, 5 mmol). An orange red product was obtained (2.90 g, 4.63 mmol, 93%). Orange crystals were obtained by the evaporation of CH_3_CN/DMF (1:4) at room temperature. ^1^H NMR (500 MHz, DMSO-d_6_, ppm): 8.40 (s, 1H, N*H*), 8.37 (s, 2H, Core*H*), 8.07 (d, 1H, Core*H*), 7.29 (t, 4H, Ar*H*), 7.15–7.19 (m, 4H, Ar*H*), 6.89 (t, 1H, Ar*H*); ^13^C NMR (300 MHz, DMSO-d_6_/TFA-d, ppm): 150.25, 139.31, 138.73, 138.26, 137.03, 135.74, 134.76, 134.49, 134.39, 134.28, 132.77, 128.94, 127.56, 126.95, 126.02, 124.01, 120.25, 116.48, 112.72; FT-IR (KBr pellet, cm^−1^): 1712(s), 1592(m), 1517(m), 1494(m), 1451(w), 1327(m), 1309(m); HRMS(APCI) m/z calcd: 627.2032 [M+H]^+^, found 627.2025; Anal. calcd for C_40_H_26_N_4_O_4_ (%): C 76.67; H 4.18; N 8.94, found: C 77.14; H 4.16; N 8.87.

**N, N′-bis[*p*-phenylamino(phenyl)]-pyromellitic diimide (DPD) 4**

A similar method as described above was employed by the substitution of naphthalene-1,4,5,8-tetracarboxylic dianhydride with pyromellitic dianhydride (1.09 g, 5 mmol). An orange product was obtained (2.56g, 4.65 mmol, 93%). ^1^H NMR (500 MHz, DMSO-d_6_, ppm): 8.40 (s, 1H, N*H*), 8.32 (s, 1H, Core*H*), 7.27-7.33 (m, 4H, Ar*H*), 7.15–7.19 (m, 4H, Ar*H*), 6.90 (t, 1H, Ar*H*); ^13^C NMR (300 MHz, DMSO-d_6_/TFA-d, ppm): 140.90, 139.50, 136.70, 134.44, 134.33, 132.46, 127.63, 126.02, 124.00, 123.69, 120.24, 116.48, 112.72; FT-IR (KBr pellet, cm^−1^): 1720(s), 1603(m), 1526(m), 1504(w), 1494(w), 1469(w), 1453(w), 1320(m), 1307(m); HRMS(APCI) m/z calcd: 551.1719 [M+H]^+^, found 551.1721; Anal. calcd for C_34_H_22_N_4_O_4_ (%): C 74.17; H 4.03; N 10.18, found: C 74.18; H 3.94; N 10.06.

**N, N′-bis[*p*-phenylamino(phenyl)]-bicyclo[2.2.2]oct-7-ene-2,3,5,6-tetracarboxylic diimide**

**(DCTD) 5**

A similar method as described above was employed by the substitution of naphthalene-1,4,5,8-tetracarboxylic dianhydride with bicyclo[2.2.2]oct-7-ene-2,3,5,6-tetracarboxylic dianhydride (1.24 g, 5 mmol). A light pink product was obtained (2.48 g, 4.27 mmol, 86%). Light pink crystals were obtained by the evaporation of CH_3_CN at room temperature. ^1^H NMR (500 MHz, DMSO-d_6_, ppm): 8.32 (s, 1H, N*H*), 7.26 (t, 2H, Ar*H*), 7.09–7.11 (d-d, 4H, Ar*H*), 6.96 (d, 2H, Ar*H*), 6.87 (t, 1H, Ar*H*); 6.28 (t, 1H, ene*H*), 3.52 (s, 1H, R_3_C*H*), 3.37 (s, 2H, (C = O)C*H*); ^13^C NMR (300 MHz, DMSO-d_6_/TFA-d, ppm): 140.03, 138.58, 136.59, 135.02, 134.32, 134.21, 132.53, 127.73, 125.98, 124.02, 120.25, 116.49, 112.72, 46.63, 37.76; FT-IR (KBr pellet, cm^−1^): 1705(s), 1597(m), 1520(m), 1500(m), 1321(m), 1303(m); HRMS(ESI) m/z calcd: 581.2189 [M+H]^+^, found 581.2209; Anal. calcd for C_36_H_28_N_4_O_4_ (%): C 74.47; H 4.86; N 9.65, found: C 74.92; H 4.66; N 9.58.

**N-*p*-phenylamino(phenyl)-1,8-naphthalic monoimide (PNM) 6**

A similar method as described above was employed by the substitution of naphthalene-1,4,5,8-tetracarboxylic dianhydride with 1,8-naphthalene anhydride (0.99 g, 5 mmol). A yellow product was obtained (1.13 g, 3.10 mmol, 62%). ^1^H NMR (500 MHz, DMSO-d_6_, ppm): 8.49-8.51 (m, 4H, Core*H*), 8.32 (s, 1H, N*H*), 7.90 (t, 2H, Core*H*), 7.28 (t, 2H, Ar*H*), 7.15–7.20 (m, 6H, Ar*H*), 6.87 (t, 1H, Ar*H*); ^13^C NMR (300 MHz, DMSO-d_6_/TFA-d, ppm): 140.77, 140.35, 140.12, 136.87, 135.63, 135.07, 134.32, 134.20, 132.08, 130.88, 127.98, 126.04, 124.41, 124.01, 120.25, 116.48, 112.71; FT-IR (KBr pellet, cm^−1^):1697(m), 1658(s), 1625(w), 1608(w), 1589(s), 1535(m), 1510(m), 1494(m), 1342(m), 1329(m); HRMS(ESI) m/z calcd: 365.1290 [M+H]^+^, found 365.1304; Anal. calcd for C_24_H_16_N_2_O_2_ (%): C 79.11; H 4.43; N 7.69, found: C 79.21; H 4.29; N 7.64. 

## 3. Results and Discussion

Diimide compounds **1**–**5** and monoimide compound **6** were synthesized by the condensation of *p*-phenylamino(phenyl)amine with dianhydrides/anhydride. ^1^H NMR and ^13^C NMR spectra along with HRMS spectra either by APCI or ESI confirmed target compounds (Figures S1–S18). For example, the NH proton positions in **1**–**6** were identified as singlet peaks in DMSO-d_6_ between 8.32 and 8.40 ppm on a 500 MHz nuclear magnetic resonance spectrometer. Meanwhile, experimental values of mass-to-charge ratio (m/z, [M+H]^+^) for each compound were obtained within experimental errors. In addition, single crystal structures of compounds **1**, **3**, and **5** were also obtained. Structural analyses are discussed in Section 3.1.

### 3.1. Description of Crystal Structures

**Figure 1:**
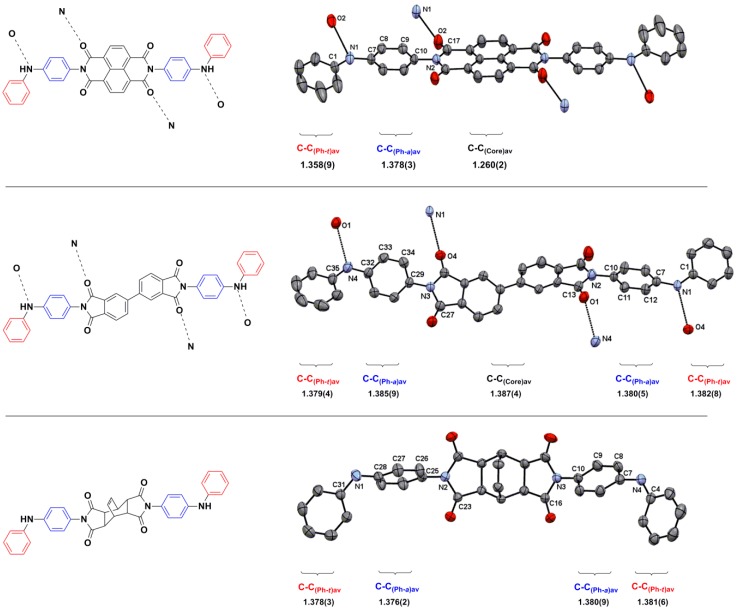
Schematic diagrams of intermolecular N···O hydrogen bonding interactions that appeared in diimide compounds for **1** (top left), **3** (middle left), and **5** (bottom left). ORTEP structures of **1** (top right), **3** (middle right), and **5** (bottom right) were presented with intermolecular N···O hydrogen bonding interactions. Solvent DMF molecules in **1** were deleted for clarity. Ph-*t* represented the terminal phenyl unit and Ph-*a* represented the adjacent phenyl unit relative to core.

**Figure 2:**
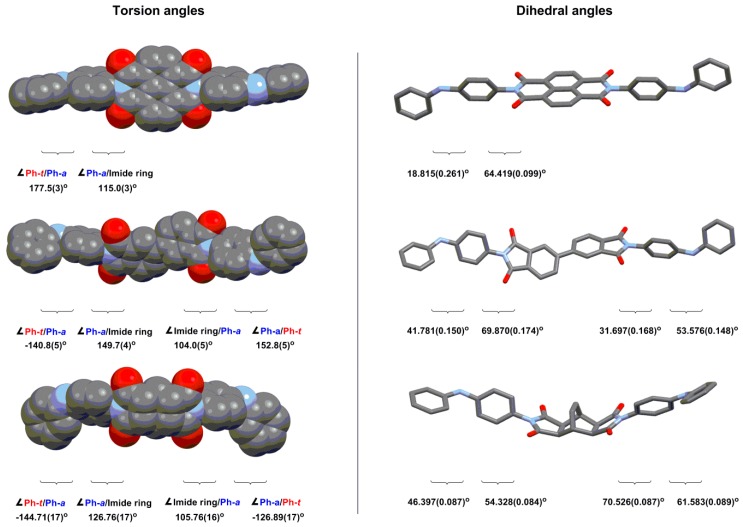
Space filling diagrams (left) with listed torsion angles and dihedral angles (right) were presented. Top left and right diagrams are for **1**, middle left and right diagrams are for **3**; bottom left and right are for **5**.

Crystallographic data for **1**, **3**, and **5** are summarized in Table 1 and selected bond lengths and torsion angles are in Table 2. Compound **1** crystallized in monoclinic space group P2_1_/n with solvent DMF molecules embedded in the lattice. As shown in Figure 1 and Figure 2, the crystal structure showed one rigid conjugated core and two *p*-phenylamino(phenyl) units interacting with neighbor molecules through intermolecular N···O hydrogen bonds. No interactions were observed for solvent DMF molecules in the solid state. Selected C–N bond lengths in **1** (Figure 1 and Table 2) showed a longer distance for C10–N2 (1.444(3) Å) than both C1–N1 (1.385 (4) Å) and C7–N1 (1.389 (3) Å) due to twisted Ph-*a*/Imide ring angles and electron withdrawing imide groups. Meanwhile, the average bond length of carbon–carbon in Ph-*t* (C–C(Ph-*t*)_av_)—Ph-*t* represents the terminal phenyl unit relative to core—was 1.358(9) Å shorter than the average bond length of carbon–carbon that was 1.378 (3) Å in Ph-*a* (C–C(Ph-*a*)_av_). Ph-*a* represents the adjacent phenyl unit was relative to core. The average carbon–carbon bond length in core, C–C(core)_av_, was 1.260(2) Å relatively shorter than either C–C(Ph-*t*)_av_ or C–C(Ph-*a*)_av_. 

**Figure 3:**
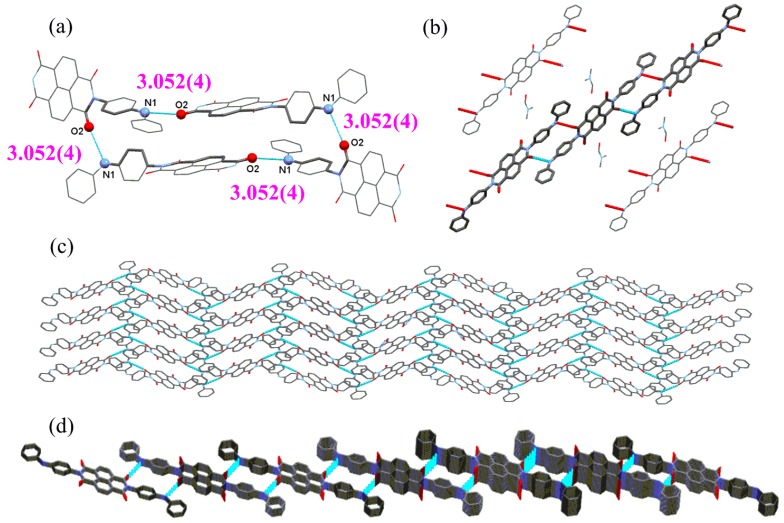
Perspective views for **1** formed by intermolecular N···O hydrogen bonds (**a–d**) are presented. A large pore was formed by four molecules with equivalent one type of N···O hydrogen bonds (**a**). Views of embedded solvent DMF molecules (**b**), solvent free 2D network (**c**), and channels (**d**) are presented. The greenish blue dashed line represents the intermolecular N···O hydrogen bonds. The red dashed line represents hanging contacts.

The space filling diagram of **1** showed twisted connections between the imide rings and *p*-phenylamino(phenyl) units. The torsion angle of C9-C10-N2-C17 between the Ph-*a* and imide ring, ∠Ph-*a*/Imide ring, was 115.0(3)°. A dihedral angle of 64.419(0.099)° between ∠Ph-*a*/Imide ring was also obtained. In contrast, the torsion angle of C8-C7-N1-C1, ∠Ph-*t*/Ph-*a*, was 177.5(3)°. Together with a dihedral angle of 18.815(0.261)° between ∠Ph-*t*/Ph-*a*, these values indicate the co-planarity for Ph-*t* and Ph-*a* (Figure 2 and Table 2). Compound **1** was linked together through one type of intermolecular N···O hydrogen bond (3.052(4) Å, ∠N1H1O2 = 155.1°) to form a large pore size 2D channel (Figure 3).

Compound **3** crystallized in the triclinic space group P-1. As shown in Figure 1 and Figure 2, the crystal structure showed one twisted core and two *p*-phenylamino(phenyl) units interacting with neighbor molecules through intermolecular N···O hydrogen bonds. Selected C–N bond lengths in **3** (Figure 1 and Table 2) showed longer distances for C10–N2 (1.436(5) Å) and C29–N3 (1.440(5) Å) than C1–N1 (1.402 (5) Å), C7–N1 (1.389 (5) Å), C35–N4 (1.417 (6) Å), and C32–N4 (1.406 (5) Å) in *p*-phenylamino(phenyl) units due to similar reasons as compound 1. In addition, lower symmetry was observed in **3** than in **1**. Interestingly, the average bond length of 1.387(4) Å for C–C(core)_av_ was comparable to both C–C(Ph-*t*)_av_ (1.379(4) and 1.382(8) Å) and C–C(Ph-*a*)_av_ (1.385(9) and 1.380(5) Å).

**Figure 4:**
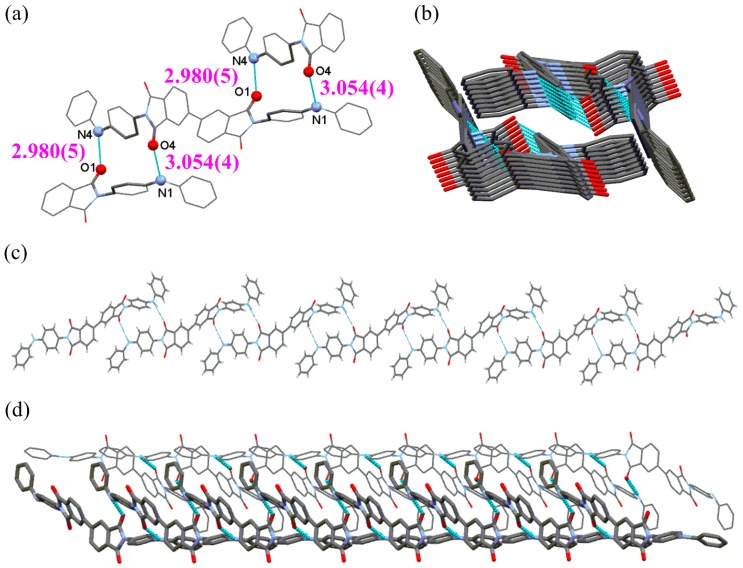
Perspective views for **3** formed by intermolecular N···O hydrogen bonds (**a**–**d**) are presented. A small pore was formed by two molecules with non-equivalent two types of N···O hydrogen bonds (**a**). Views of a single 1D chain (**c**), and double chains (**b**,**d**) for 3 are presented. The greenish blue dashed line represents intermolecular N···O hydrogen bonds.

The space filling diagram of **3** showed a highly twisted configuration. The dramatically decreased torsion angles of ∠Ph-*t*/Ph-*a*, with 152.8(5)°and -140.8(5)°compared to 177.5(3)° in **1**, were observed for **3** (Figure 2 and Table 2). Furthermore, the torsion angles of ∠Ph-*a*/Imide ring were either slightly decreased to 104.0(5)° or significantly increased to 149.7(4)° due to the less conjugated core in a free rotation mode. In other words, the increased dihedral angles of ∠Ph-*t*/Ph-*a*, with 53.576(0.148)° and 41.781(0.150)° compared to 18.815(0.261)° in **1**, were observed for **3**. Dihedral angles of ∠Ph-*a*/Imide ring were either slightly decreased to 31.697(0.168)° or significantly increased to 69.870(0.174)°. Besides, compound **3** was linked together through two types of intermolecular N···O hydrogen bonds (2.980(5) Å, ∠N4H4AO1 = 145.3° and 3.054(4) Å, ∠N1H1O4 = 162.2°) to form a small pore size 1D chain (Figure 4).

Compound **5** crystallized in the monoclinic space group P2_1_/c. As shown in Figure 1, the structure contained a non-conjugated core and two *p*-phenylamino(phenyl) units without intermolecular N···O hydrogen bonds. Selected C–N bond lengths in **5** showed longer distances for C25–N2 (1.434(2) Å) and C10–N3 (1.435(2) Å) than C31–N1 (1.406 (2) Å), C28–N1(1.408 (2) Å), C4–N4 (1.391 (2) Å), and C7–N4 (1.398 (2) Å) in *p*-phenylamino(phenyl) units (Figure 1 and Table 2). The average bond lengths of C–C(Ph-*t*)_av_ (1.378(3) and 1.381(6) Å) and C–C(Ph-*a*)_av_ (1.376 (2) Å and 1.380 (9) Å) in **5** were comparable to the corresponding average carbon–carbon bond lengths in **1** and **3**.

The space filling diagram of **5** showed twisted connections between the non-conjugated core and *p*-phenylamino(phenyl) units. The torsion angles of ∠Ph-*t*/Ph-*a* with −144.71(17)° (a dihedral angle of 46.397(0.087)°) and (−126.89(17)°(a dihedral angle of 61.583(0.089)°) were also significantly decreased contrast to 177.5(3)° (a dihedral angle of 18.815(0.261)°) in 1 (Figure 2 and Table 2). On the contrary, the torsion angles and dihedral angles of ∠Ph-*a*/Imide ring were almost equally changed by 10° either clockwise or anticlockwise. The highly bent non-conjugated core was likely to block the approach of neighbor molecules, which prevented the existence of intermolecular N···O hydrogen bonding interactions in **5**.

### 3.2. Infrared Spectra

IR (infrared) spectra for **1**–**6** were recorded on a Bruker MPA spectrometer between 400 and 4000 cm^−1^ (KBr pellets). For compound **1**, the observed semicircle stretch modes of para-substituted benzene ring appeared at 1509 and 1409 cm^−1^, while the terminal benzene ring stretches appeared at 1496 and 1448 cm^−1^ and the conjugated carbon–carbon stretch for the NDI core was observed at 1581 cm^−1^. The strong characteristic imide stretch modes were identified at 1712 and 1666 cm^−1^. The carbon–nitrogen stretch modes were observed at 1328 and 1345 cm^−1^ [34,42]. Similar assignments for 2−6 were also observed in Figures S19 and S20.

### 3.3. Thermogravimetric Analyses

TGA data for **1**–**6** showed two stages for weight losses. The first stage experienced rapid minor weight losses below 100°C that were assigned to lost solvents from corresponding solid samples, evidenced by steady curves after 100°C. Sudden weight losses started at 395°C for 5 and 391 °C for **6** during the second stage, that were assigned to broken bonds of the Ph-*a* and imide ring, which further evaporated to almost nothing. Decomposition temperatures for **1**–**4** were at higher temperatures of 453 °C for **1**, 501 °C for **2**, 517 °C for **3**, and 463 °C for **4**. Interestingly, unknown polymers, possibly high thermal stable polyimides, were formed, as evidenced by the steady curves (Figure S21). Excellent thermal stabilities under the N_2_ stream were also supported by the existence of hydrogen bonds. Based on the solved crystal structures of **1**, **3**, and **5**, a conclusion was drawn that thermal stabilities for **1** and **3** were enhanced by hydrogen bonds, but this was not the case for **5**, due to the absence of such interactions. Particularly, the two types of short hydrogen bonds were responsible for the most stable compound **3**.

### 3.4. UV-Vis Absorption and Fluorescence Spectra

**Figure 5:**
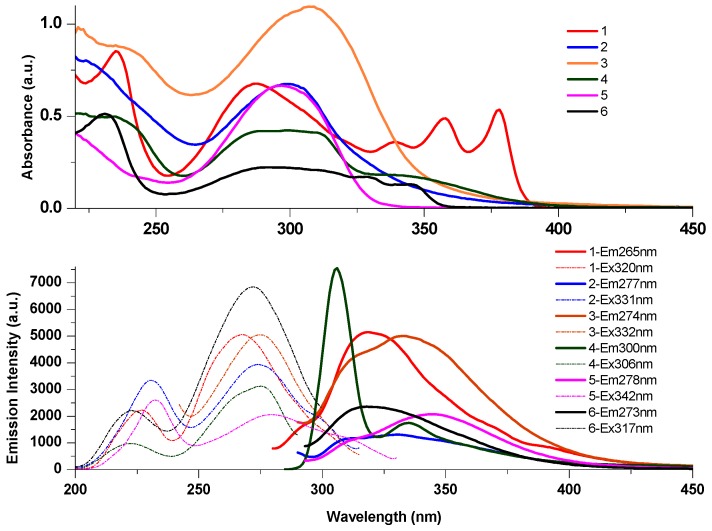
UV-vis absorption spectra (top), excitation spectra (bottom left, dashed line) and emission spectra of **1**–**6** (bottom right, solid line) in THF (2 × 10^−5^ M) were recorded at room temperature in air. Excitation wavelengths of 265 nm for **1**, 277 nm for **2**, 274 nm for **3**, 300 nm for **4**, 278 nm for **5**, and 273 nm for **6** were applied to obtain emission spectra.

Steady-state UV-vis absorption and fluorescence emission spectra for **1**–**6** were recorded in THF, DCM, EtOH, and CH_3_CN, respectively. UV-vis absorption spectra for **1**–**6** in THF showed that all peaks appeared below 400 nm (Figure 5). Major absorption peaks appeared at 287 nm for **1**, 299 nm for **2**, 307 nm for **3**, 300 nm for **4**, 297 nm for **5**, and 291 nm for **6**. These peaks were assigned to π–π* transitions from conjugated aromatic units, while the rest of the lower energy peaks in the spectra were assigned to ICT transitions [43]. Similar absorption peaks recorded in DCM, EtOH, and CH_3_CN were also obtained (Figures S22–S25).

For compound **1**, an emission peak appeared at 319 nm under the excitation of 265 nm, which matched well with the corresponding excitation peak at 267 nm (Figure 5). Similarly, an emission peak appeared at 318 nm under the excitation of 273 nm for 6. In contrast, a shoulder peak appeared at 312 nm for **2**, except for the major emission peak at 331 nm. Shoulder emission peaks at 311 nm for 3, 335 nm for **4**, and 306 nm for **5**, along with major emission peaks at 333 nm, 306 nm, and 342 nm, were observed accordingly.

**Figure 6:**
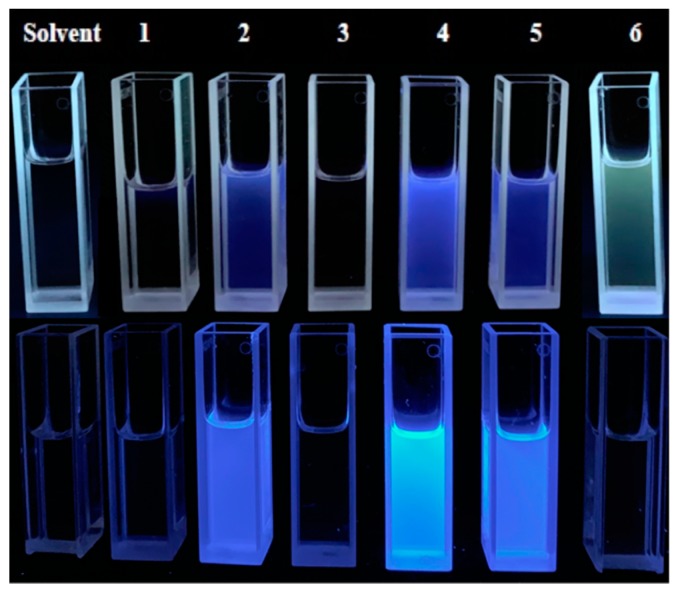
Emission photographs of solvent THF (most left) and **1**–**6** in THF, excited at 254 nm (top) and 365 nm (bottom), were recorded at room temperature in air.

**Figure 7:**
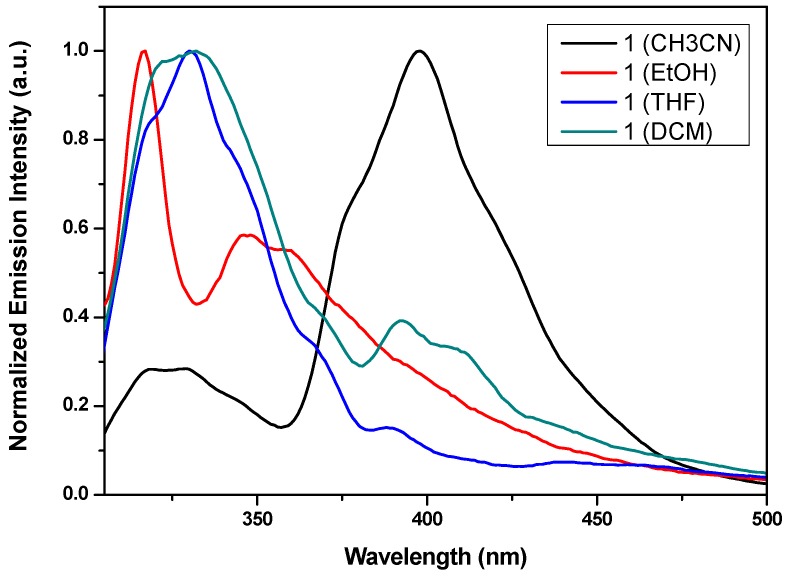
Normalized emission spectra of **1** were excited at 286 nm in CH_3_CN (black), EtOH (red), THF (blue), and DCM (greenish blue) at room temperature in air.

The structured emission spectra for **1**–**6** indicated a function of a UV filter. The more harmful UV lights from 265 nm to 300 nm, UV-C to UV-B, were absorbed by such compounds, giving lights from 306 nm to 342 nm, in the range of UV-B to UV-A [44]. Bright deep-blue or violet-blue emissions for **2**, **4**, and **5** in diluted THF solutions were observed under excitations of 365 nm and 254 nm at room temperature in air (Figure 6). Solvent effects on the steady-state emission spectra for **1**–**6** in CH_3_CN, EtOH, THF, and DCM at room temperature in air were also studied (Figure 7, Figures S26–S31). For 1, major fluorescence peaks were red-shifted from 332 nm in DCM to 398 nm in CH_3_CN with the increasing of polarity. An emission peak at 317 nm in EtOH was sharp and resolved compared to the broad and overlapped peaks in other solvents. A similar trend was also observed for **2**, **3**, **4** and **6**. For **5**, the emission profile was quite different from **1**, **2**, **3**, **4**, and **6**, also matching with polarity effect. The dramatic difference was tentatively attributed to the existence of a non-conjugated core only in **5**.

Therefore, aromatic conjugation greatly contributed to emission profiles and peak positions. Moreover, the characterized crystal structure for 5 revealed the absence of intermolecular N···O hydrogen bonds, which might be responsible for the decreased chances of forming types of hydrogen-bonded superstructures. To some extent, the existence of hydrogen bonds led to complicated emission profiles, non-radiative decay, energy transfer, and quenched fluorescence [45,46,47,48,49]. For **1** and **3**, intermolecular N···O hydrogen bonds were observed in characterized structures that could induce weak or quenched fluorescence in solid state. In solution state, freely moving molecules still have opportunities to form hydrogen bonds due to the existence of N–H and imide units in **1**–**6**.

Fluorescence spectra for **1**–**6** in the solid state were also obtained under excitations of 250 nm and 280 nm at room temperature in air (Figures S32–S34). Accordingly, emission peaks appeared at 440 nm, 400 nm, and 360 nm for **1**, **4**, and **5**. It is a common phenomenon that aggregation-caused quenching (ACQ) occurs in conjugated molecules due to intermolecular interactions within a short range, which leads to the enhancement of non-radiative decay pathways [28,50]. Therefore, the less conjugated compound **5** without intermolecular N···O hydrogen bonds in solid state was brighter than the intermolecular N···O hydrogen bonded compounds of **1** and **3**. In addition, PLQYs of **2** (PLQY = 4.69% in solid; PLQY = 7.67% in THF solution), **4** (PLQY = 5.43% in solid; PLQY = 1.28% in THF solution), and **5** (PLQY = 6.39% in solid; PLQY = 0.74% in THF solution) were obtained at room temperature in air. A potential application of **2**, **4**, and **5** can be considered for deep-blue or violet-blue emitters in solid state according to studies of blue OLEDs [43,51,52]. The exploration of being probes in solution state is also interesting.

### 3.5. Differential Pulse Voltammetry (DPV) and Cyclic Voltammogram (CV)

**Figure 8:**
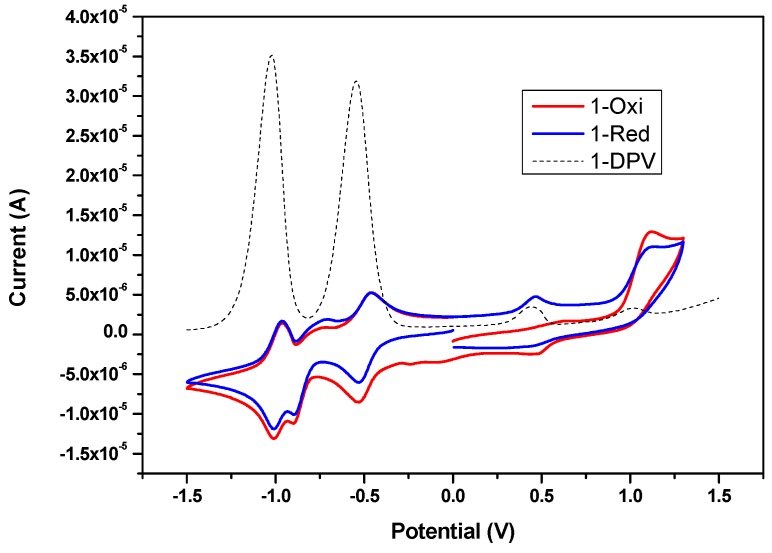
Differential pulse voltammetry (DPV) in the black dashed line and a cyclic voltammogram (CV) of **1** were measured in DMF with 0.1 M TBAPF_6_. The CV of 1-Oxi in the red solid line indicates that oxidation was run first and the 1-Red in the blue solid line indicates that reduction was run first. Pt wire, Pt disk, and Ag/AgCl were used for measurements with a scan rate of 0.1 Vs^−1^.

Peaks from DPV at 0.47 V and 1.01 V, together with oxidation peaks by CV at around 0.50 V and 1.10 V for compound **1** suggest the formation of diphenylbenzidene after two subsequent 1 e oxidation reactions. E_1/2_ values at −0.50 V and −1.00 V were consistent with DPV peaks appearing at −0.55 V and −1.02 V for the two 1 e reductions of the NDI core (Figure 8) [34,35,37,38]. Similarly, two 1 e reductions of the 4,4′-biphthalic core in **3** were observed at potentials of −1.27 V and −1.17 V by DPV, which were consistent with E_1/2_ values at −1.33 V and −1.14 V by CV. On the contrary, only one 1 e reduction of the 1,8-naphthalene unit in **6** at −1.27 V by DPV with an E_1/2_ value at −1.24 V by CV were observed (Figures S35–S39). Meanwhile, oxidation and reduction potentials for **1**–**6** were not obviously affected either by oxidation run first or reduction run first.

## 4. Conclusions

In summary, a series of diimide and monoimide compounds condensed with *p*-phenylamino(phenyl)amine were successfully synthesized and confirmed. Single crystal structures showed one type of intermolecular N···O hydrogen bond in **1**, forming 2D large pore size channels, and two types of intermolecular N···O hydrogen bonds in **3**, forming a 1D chain. All compounds showed high thermal stabilities. Particularly, the two types of intermolecular N···O hydrogen bonds in **3** play a vital role for thermal stability property. Fluorescence studies conducted both in solution and in solid states indicated that intermolecular N···O hydrogen bonding interactions quenched fluorescence. Diimide compounds **2**, **4**, and **5** were potential deep-blue or violet-blue emitters in solid state. The exploration of such compounds in areas of probes, electrochromic materials, information security, and semiconductors are worthy of consideration.

## Figures and Tables

**Table 1:** Crystallographic data for **1**, **3**, and **5**.

	1	3	5
Empirical formula	C_38_H_24_N_4_O_4_ ·2DMF	C_40_H_26_N_4_O_4_	C_36_H_28_N_4_O_4_
CCDC	1859060	1859059	1859058
Formula weight	746.80	626.65	580.62
Temperature (K)	296(2)	296(2)	296(2)
*λ* (Å)	0.71073	0.71073	0.71073
Crystal system	Monoclinic	Triclinic	Monoclinic
Space group	P2_1_/n	P-1	P2_1_/c
*a* (Å)	13.917(7)	8.3054(19)	9.457(10)
*b* (Å)	5.811(3)	12.394(3)	25.27(3)
*c* (Å)	22.797(11)	14.874(3)	12.610(14)
*α* (°)	90	103.340(4)	90
*β* (°)	97.101(8)	91.546(4)	109.546(13)
*γ* (°)	90	94.871(4)	90
*V* (Å^3^)	1829.7(15)	1482.6(6)	2840(5)
Z	2	2	4
*D_calcd_* (mg·m^−3^)	1.356	1.404	1.358
*μ* (mm^−1^)	0.092	0.092	0.090
*F* (000)	784	652	1216
*θ* (°)	2.913–27.570	2.446–25.000	2.285–27.618
Reflections /unique	10894/4195	7779/5157	17897/6511
GOF on F^2^	0.959	0.855	1.042
R_1_^a^, *w*R_2_^b^ [*I* > 2σ(*I*)]	0.0665, 0.1697	0.0662, 0.1294	0.0432, 0.1090
R_int_	0.0621	0.0636	0.0332

^a^ R_1_=∑||F_o_|−|F_c_||/∑|F_o_|. ^b^ *w*R2 = [∑w(F_o_^2^−F_c_^2^)^2^/∑w(F_o_^2^)^2^]^½^.

**Table 2:** Selected bond lengths (Å) and torsion angles (°) for **1**, **3**, and **5.**

1		3		5	
	Bond Length		Bond Length		Bond Length
C1–N1	1.385(4)	C1–N1	1.402(5)	C31–N1	1.406(2)
C7–N1	1.389(3)	C7–N1	1.389(5)	C28–N1	1.408(2)
		C35–N4	1.417(6)	C4–N4	1.391(2)
		C32–N4	1.406(5)	C7–N4	1.398(2)
C10–N2	1.444(3)	C10–N2	1.436(5)	C25–N2	1.434(2)
		C29–N3	1.440(5)	C10–N3	1.435(2)
C–C(Ph-*t*)_av_	1.359(0)	C–C(Ph-*t*)_av_	1.379(4)	C–C(Ph-*t*)_av_	1.378(3)
C–C(Ph-*a*)_av_	1.378(3)	C–C(Ph-*a*)_av_	1.385(9)	C–C(Ph-*a*)_av_	1.376(2)
C–C(Core)_av_	1.260(2)	C–C(Core)_av_	1. 387(9)		
		C–C(Ph-*t*)_av_	1.382(8)	C–C(Ph-*t*)_av_	1.381(6)
		C–C(Ph-*a*)_av_	1.380(5)	C–C(Ph-*a*)_av_	1.380(9)
		C–C(Core)_av_	1. 386(8)		
	Torsion Angle		Torsion Angle		Torsion Angle
C8–C7–N1–C1	177.5(3)	C12–C7–N1–C1	152.8(5)	C27–C28–N1–C31	−126.89(17)
C9–C10–N2–C17	115.0(3)	C11–C10–N2–C13	104.0(5)	C26–C25–N2–C23	105.76(16)
		C33–C32–N4–C35	−140.8(5)	C8–C7–N4–C4	−144.71(17)
		C34–C29–N3–C27	149.7(4)	C9–C10–N3–C16	126.76(17)

## Supplementary Materials

The following are available online at https://www.mdpi.com/1996-1944/12/11/1873/s1, spectroscopic data for **1**–**6** were provided as supplementary information. CCDC 1859058, 1859059, and 1859060 contain the supplementary crystallographic data for this paper. These data can be obtained free of charge via www.ccdc.cam.ac.uk/data_request/cif, or by emailing data_request@ccdc.cam.ac.uk, or by contacting The Cambridge Crystallographic Data Centre, 12 Union Road, Cambridge CB2 1EZ, UK; fax: +44 1223 336033. Figure S1: ^1^H NMR for **1** in DMSO-d_6_ recorded on a 500M Hz spectrometer at 303 K.; Figure S2: ^13^C NMR for **1** in DMSO-d_6_/TFA-d solvents recorded on a 300M Hz spectrometer at 303 K. Figure S3: ^1^H NMR for **2** in DMSO-d_6_ recorded on a 500M Hz spectrometer at 303 K. Figure S4: ^13^C NMR for **2** in DMSO-d_6_/TFA-d solvents recorded on a 300M Hz spectrometer at 303 K. Figure S5: ^1^H NMR for **3** in DMSO-d_6_ recorded on a 500M Hz spectrometer at 303 K. Figure S6: ^13^C NMR for **3** in DMSO-d_6_/TFA-d solvents recorded on a 300M Hz spectrometer at 303 K. Figure S7: ^1^H NMR for **4** in DMSO-d_6_ recorded on a 500M Hz spectrometer at 303 K. Figure S8: ^13^C NMR for **4** in DMSO-d_6_/TFA-d solvents recorded on a 300M Hz spectrometer at 303 K. Figure S9: ^1^H NMR for **5** in DMSO-d_6_ recorded on a 500M Hz spectrometer at 303 K. Figure S10: ^13^C NMR for **5** in DMSO-d_6_/TFA-d solvents recorded on a 300M Hz spectrometer at 303 K. Figure S11: ^1^H NMR for **6** in DMSO-d_6_ recorded on a 500M Hz spectrometer at 303 K. Figure S12: ^13^C NMR for **6** in DMSO-d_6_/TFA-d solvents recorded on a 500M Hz spectrometer at 303 K. Figure S13: APCI for **1**. Figure S14: APCI for **2**. Figure S15: APCI for **3**. Figure S16: APCI for **4**. Figure S17: ESI for **5**. Figure S18: ESI for **6**. Figure S19: FT-IR spectra of **1**, **2**, and **3** (KBr pellets). Figure S20: FT-IR spectra of **4**, **5**, and **6** (KBr pellets). Figure S21: TGA curves for **1–6**. Figure S22: UV-vis spectra for **1–6** in DCM. Figure S23: UV-vis spectra for **1–6** in THF. Figure S24: UV-vis spectra for **1–6** in EtOH. Figure S25: UV-vis spectra for **1–6** in CH_3_CN. Figure S26: Normalized emission spectra of **2** were excited at 290 nm in CH_3_CN (black), EtOH (red), THF (blue), and DCM (greenish blue), respectively at room temperature in air. Figure S27: Normalized emission spectra of **3** were excited at 286 nm in CH_3_CN (black), EtOH (red), THF (blue), and DCM (greenish blue), respectively at room temperature in air. Figure S28: Normalized emission spectra of **4** were excited at 284 nm in CH_3_CN (black), EtOH (red), THF (blue), and DCM (greenish blue), respectively at room temperature in air. Figure S29: Normalized emission spectra of **5** were excited at 290 nm in CH_3_CN (black), EtOH (red), THF (blue), and DCM (greenish blue), respectively at room temperature in air. Figure S30: Normalized emission spectra of **6** were excited at 290 nm in CH_3_CN (black), EtOH (red), THF (blue), and DCM (greenish blue), respectively at room temperature in air. Figure S31: Emission photographs for **1–6** in DCM (top), EtOH (middle), and CH_3_CN (bottom) excited at 254 nm (left) and 365 nm (right), respectively at room temperature in air. Figure S32: Solid state emission spectra excited at 250 nm for **1–6** at room temperature in air. Figure S33: Solid state emission spectra excited at 280 nm for **1–6** at room temperature in air. Figure S34: Emission photographs for **1–6** in solid state with regular light (top), excited at 365 nm (middle), and 254nm (bottom), respectively at room temperature in air. Figure S35: DPV in black dashed line and CV of **2** were measured in DMF with 0.1 M TBAPF_6_. CV of 2-Oxi in red solid line indicated oxidation run first and 2-Red in blue solid line indicated reduction run first. Pt wire, Pt disk, and Ag/AgCl were used for measurements with a scan rate of 0.1 Vs^−1^. Figure S36: DPV in black dashed line and CV of **3** were measured in DMF with 0.1 M TBAPF_6_. CV of 3-Oxi in red solid line indicated oxidation run first and 3-Red in blue solid line indicated reduction run first. Pt wire, Pt disk, and Ag/AgCl were used for measurements with a scan rate of 0.1 Vs^−1^. Figure S37: DPV in black dashed line and CV of **4** were measured in DMF with 0.1 M TBAPF_6_. CV of 4-Oxi in red solid line indicated oxidation run first and 4-Red in blue solid line indicated reduction run first. Pt wire, Pt disk, and Ag/AgCl were used for measurements with a scan rate of 0.1 Vs^−1^. Figure S38: DPV in black dashed line and CV of **5** were measured in DMF with 0.1 M TBAPF_6_. CV of 5-Oxi in red solid line indicated oxidation run first and 5-Red in blue solid line indicated reduction run first. Pt wire, Pt disk, and Ag/AgCl were used for measurements with a scan rate of 0.1 Vs^−1^. Figure S39: DPV in black dashed line and CV of **6** were measured in DMF with 0.1 M TBAPF_6_. CV of 6-Oxi in red solid line indicated oxidation run first and 6-Red in blue solid line indicated reduction run first. Pt wire, Pt disk, and Ag/AgCl were used for measurements with a scan rate of 0.1 Vs^−1^.
