# Supplementary material for: Synthesis, Characterization, and Crystal Structures of Imides Condensed with p-Phenylamino(Phenyl) Amine and Fluorescence Property
Source: Materials (Basel). 2019 Jun 10;12(11):1873. doi: 10.3390/ma12111873 (PMC6600954; doi:10.3390/ma12111873)
Supplement: Supplementary file 1 [file materials-12-01873-s001.pdf]

materials

Supplementary Materials

Synthesis, Characterization, and Crystal Structures of Imides Condensed with *p*-Phenylamino(Phenyl) Amine and Fluorescence Property

Jing Zhang and Huaibo Ma *

Key Laboratory of Flexible Electronics (KLOFE), Institute of Advanced Materials (IAM), Nanjing Tech University, 30 South Puzhu Road, Nanjing 211816, China; iamzj@njtech.edu.cn
* Correspondence: iamhbma@njtech.edu.cn

Table of Contents

S1. ^{1}HNMR and ^{13}CNMR spectra..P 1–6

S2. HRMS spectra..P 7–8

S3. IR spectra..P 9

S4. TGA spectra..P 10

S5. UV-vis spectra..P 11–12

S6. Fluorescence spectra..P 13–18

S7. DPV and CV spectra..P 19–21

S1. ^{1}HNMR and ^{13}CNMR spectra

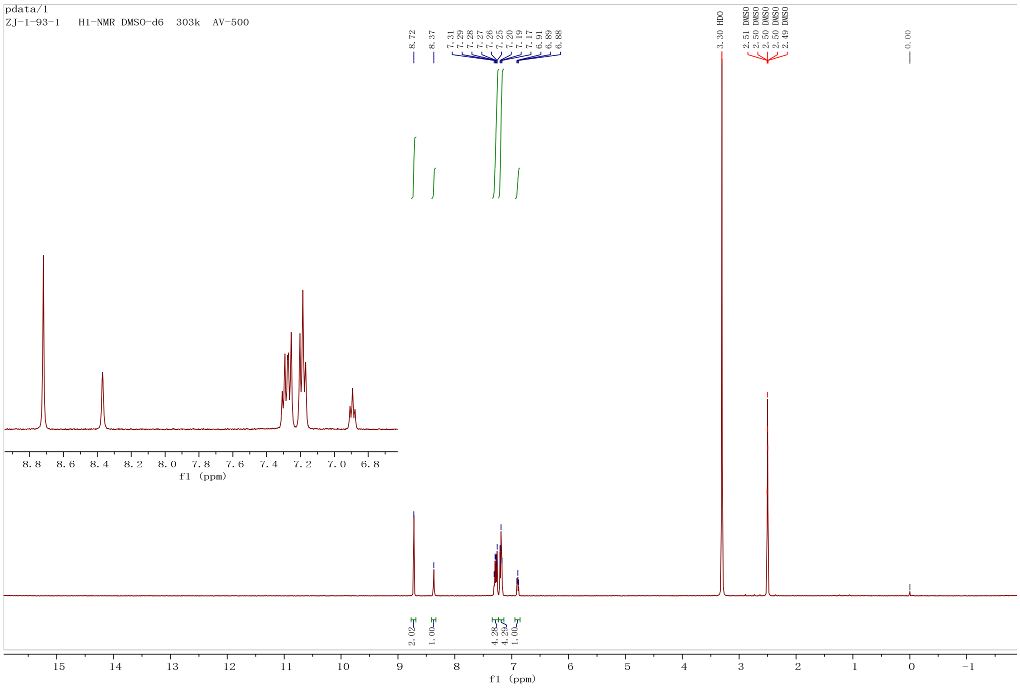

Figure S1. ^{1}H NMR for **1** in DMSO-d_6 recorded on a 500M Hz spectrometer at 303 K.

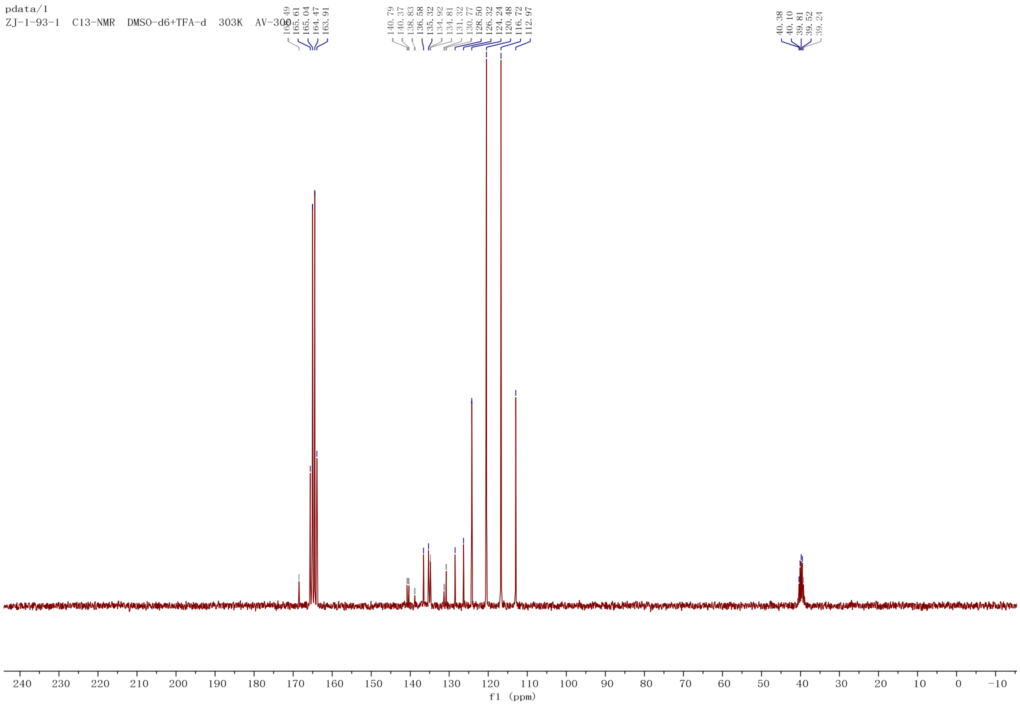

Figure S2. ^{13}C NMR for **1** in DMSO-d_6/TFA-d solvents recorded on a 300M Hz spectrometer at 303 K.

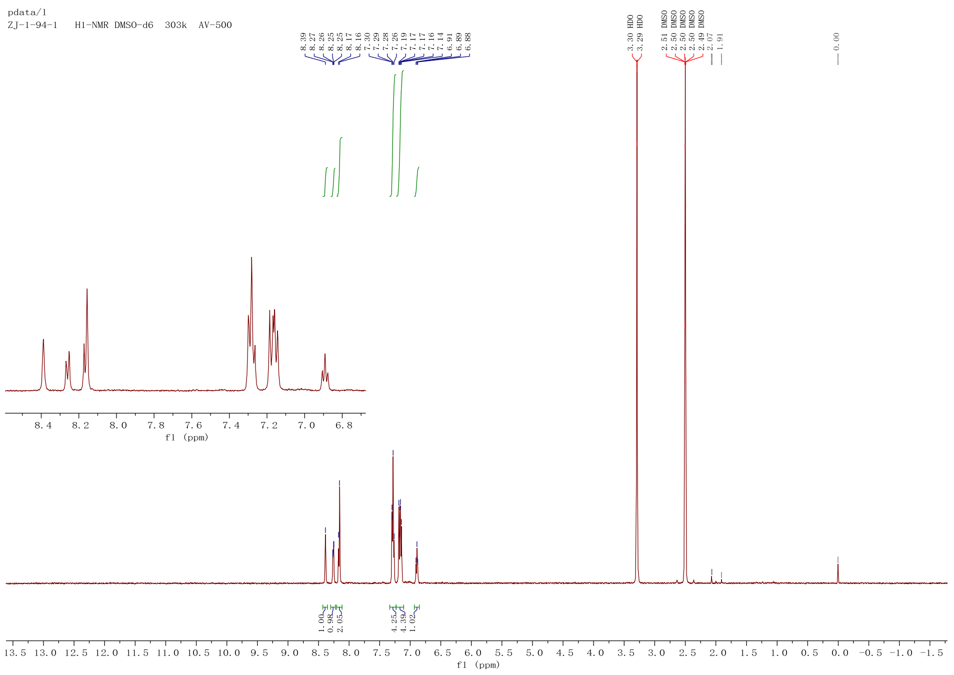

Figure S3. ^{1}H NMR for **2** in DMSO-d$_6$ recorded on a 500M Hz spectrometer at 303 K.

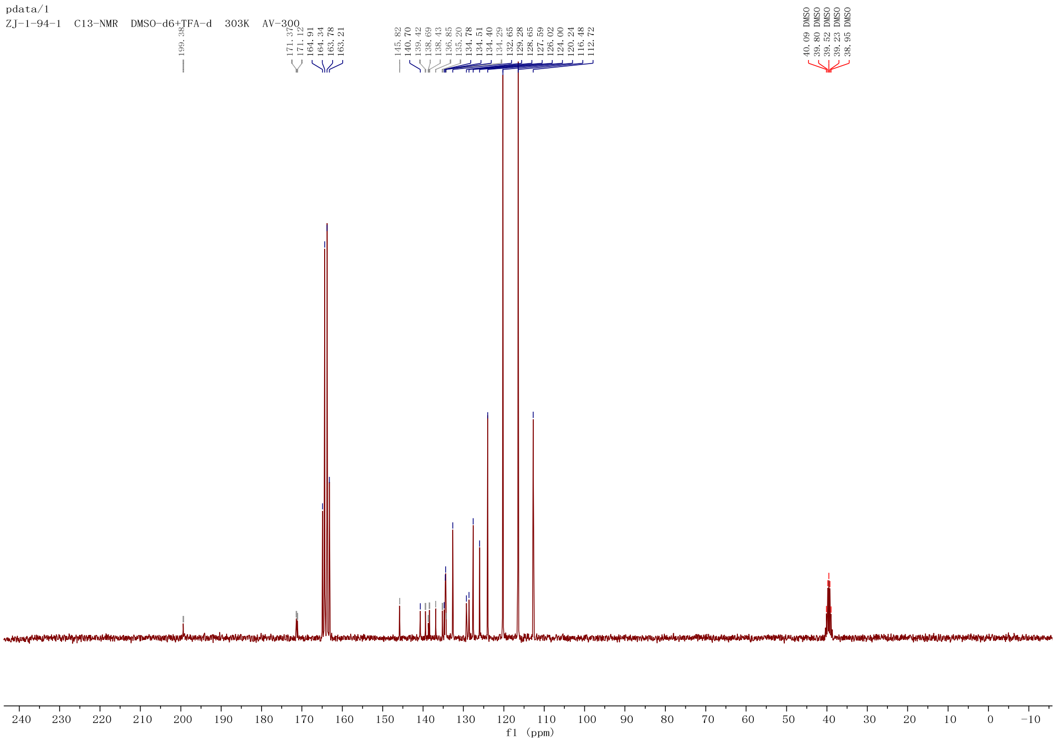

Figure S4. ^{13}C NMR for **2** in DMSO-d$_6$/TFA-d solvents recorded on a 300M Hz spectrometer at 303 K.

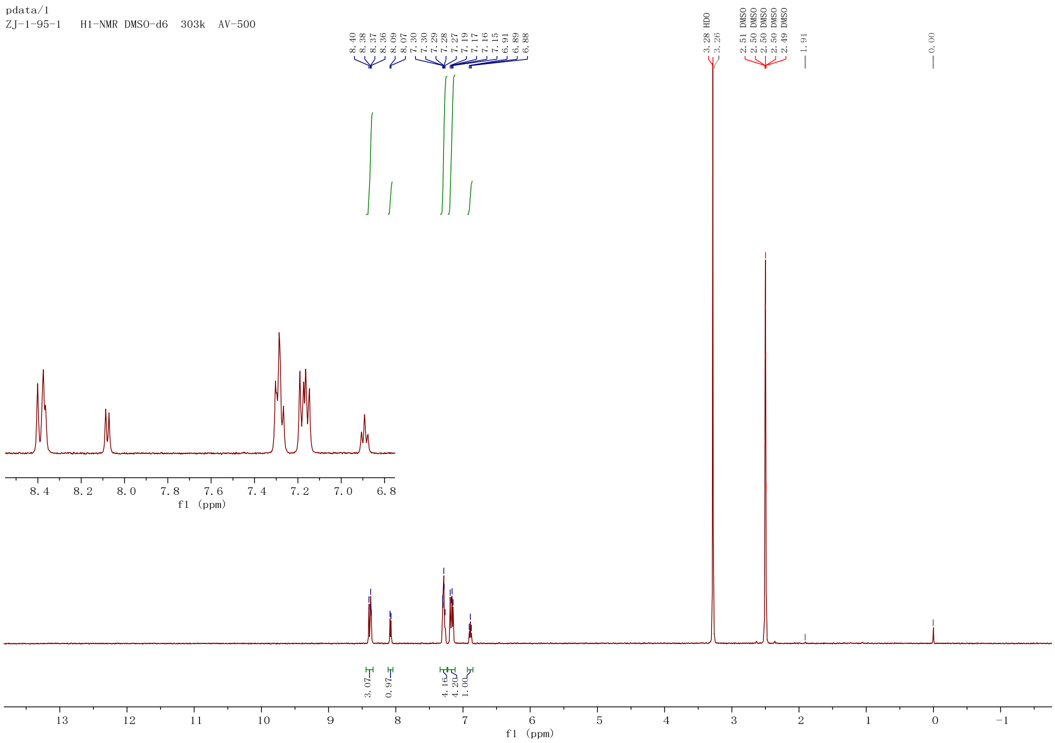

Figure S5. ^{1}H NMR for **3** in DMSO-d_6 recorded on a 500M Hz spectrometer at 303 K.

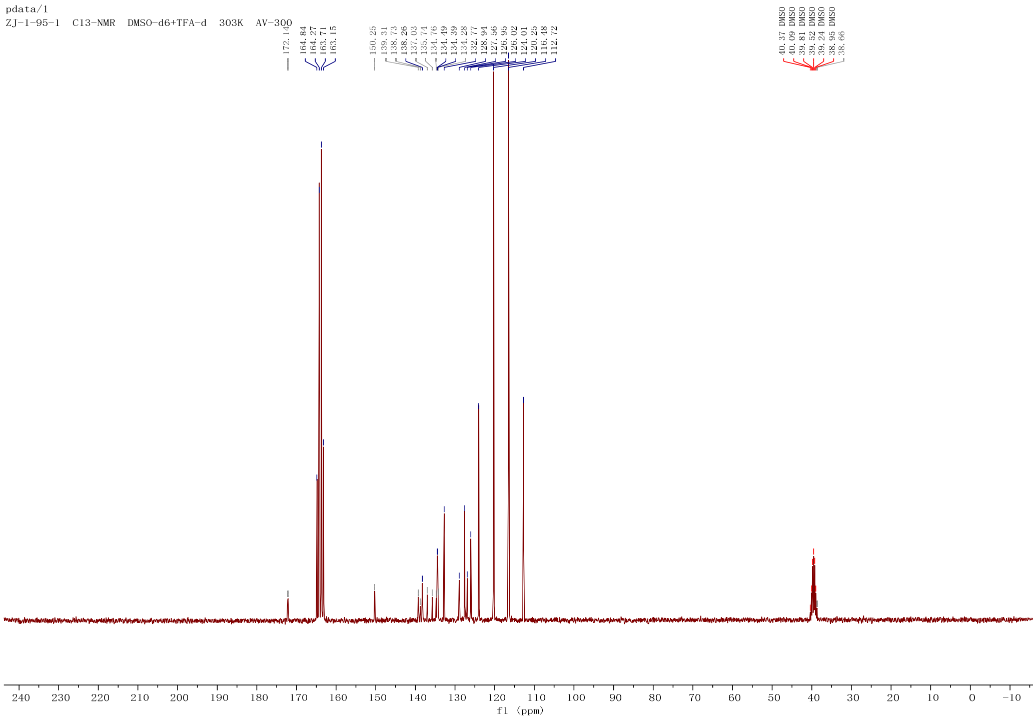

Figure S6. ^{13}C NMR for **3** in DMSO-d_6/TFA-d solvents recorded on a 300M Hz spectrometer at 303 K.

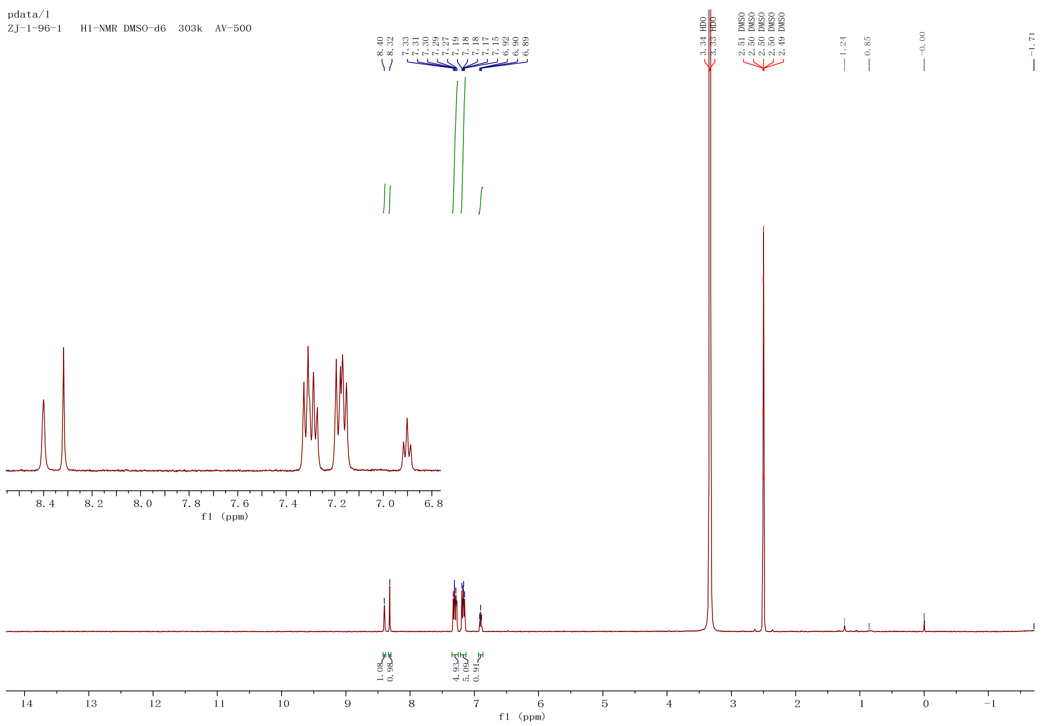

Figure S7. ^{1}H NMR for **4** in DMSO-d_6 recorded on a 500M Hz spectrometer at 303 K.

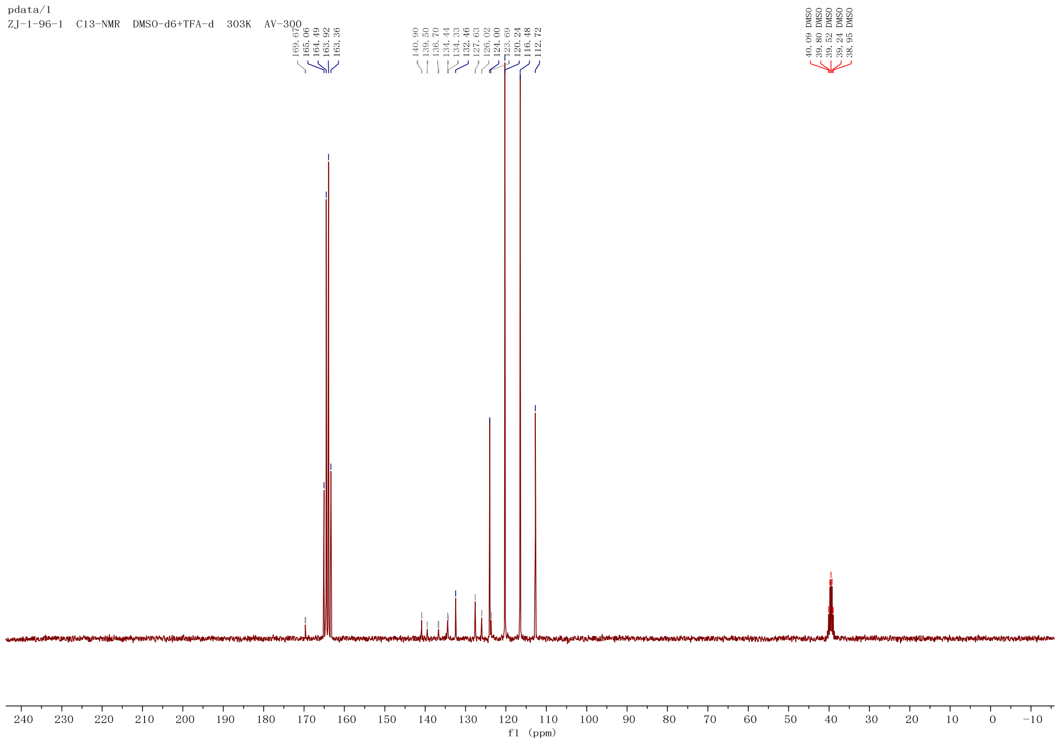

Figure S8. ^{13}C NMR for **4** in DMSO-d_6/TFA-d solvents recorded on a 300M Hz spectrometer at 303 K.

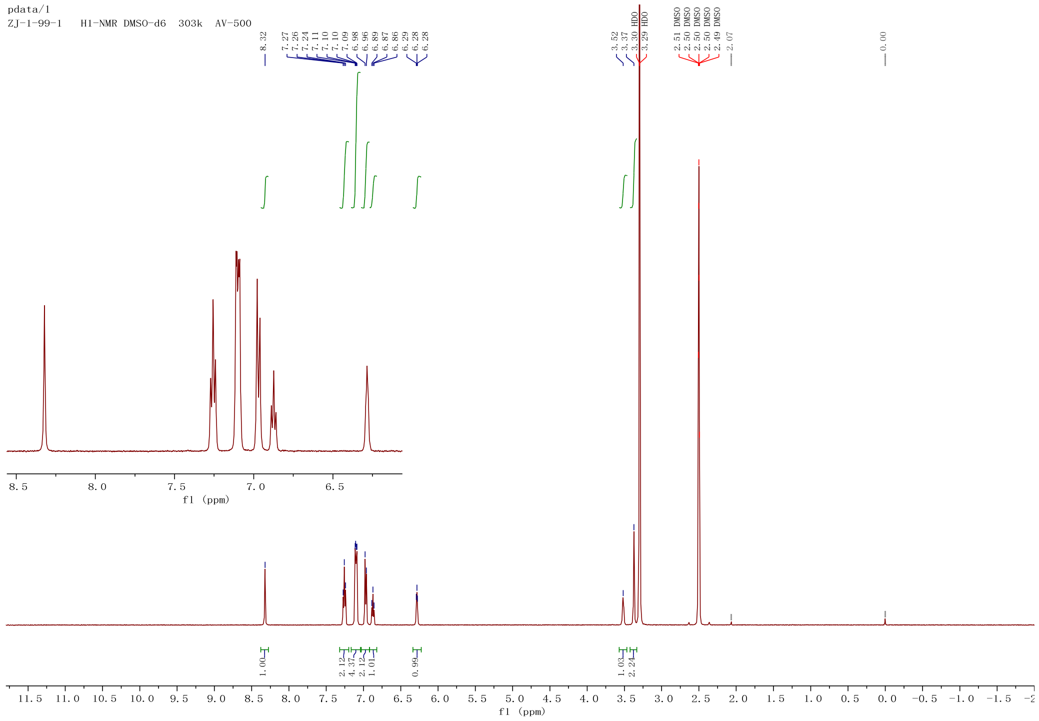

Figure S9. ^{1}H NMR for **5** in DMSO-d_6 recorded on a 500M Hz spectrometer at 303 K.

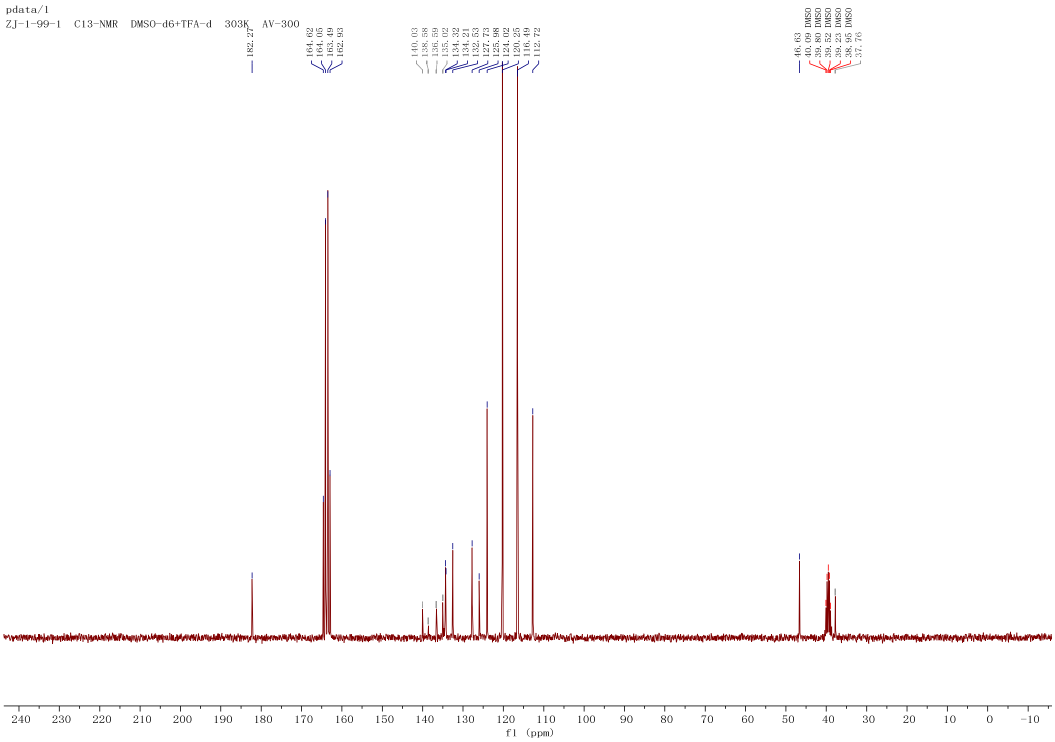

Figure S10. ^{13}C NMR for **5** in DMSO-d_6/TFA-d solvents recorded on a 300M Hz spectrometer at 303 K.

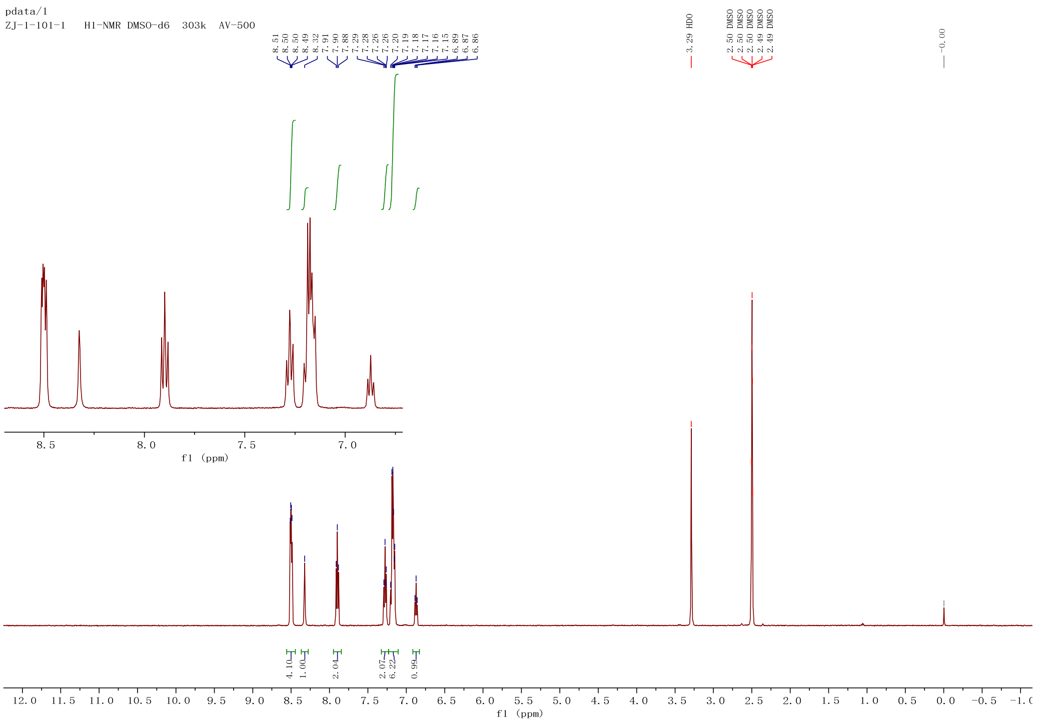

Figure S11.^{1}H NMR for **6** in DMSO-d6 recorded on a 500M Hz spectrometer at 303 K.

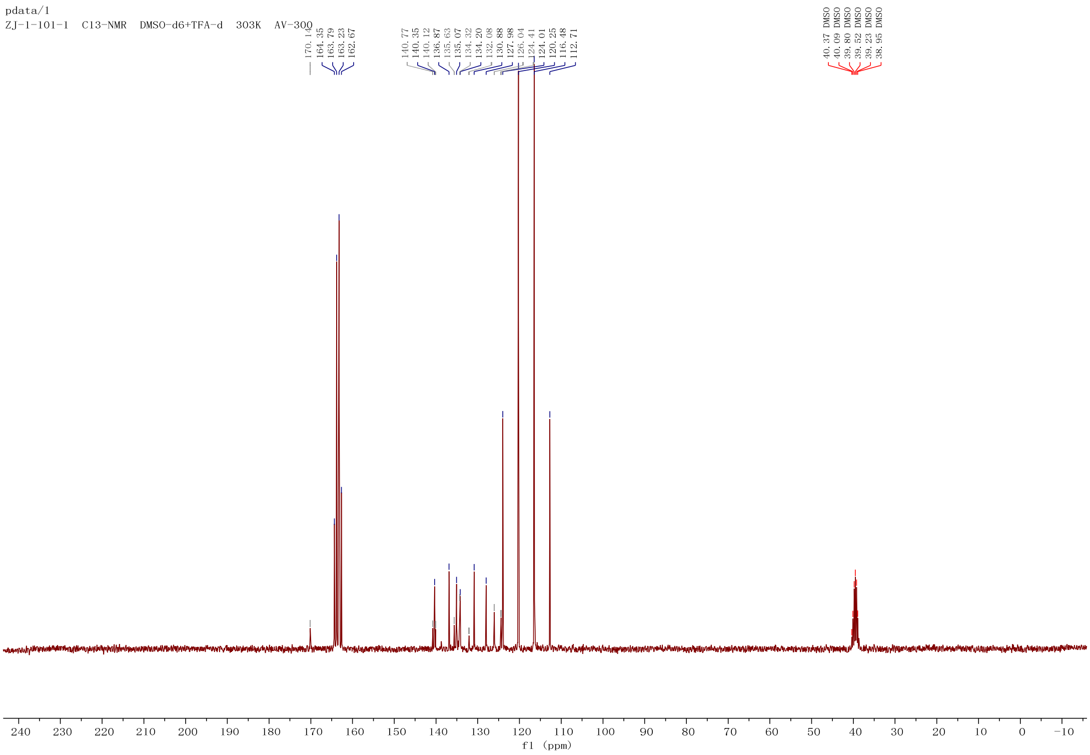

Figure S12. ^{13}C NMR for **6** in DMSO-d6/TFA-d solvents recorded on a 500M Hz spectrometer at 303 K.

S2. HRMS spectra

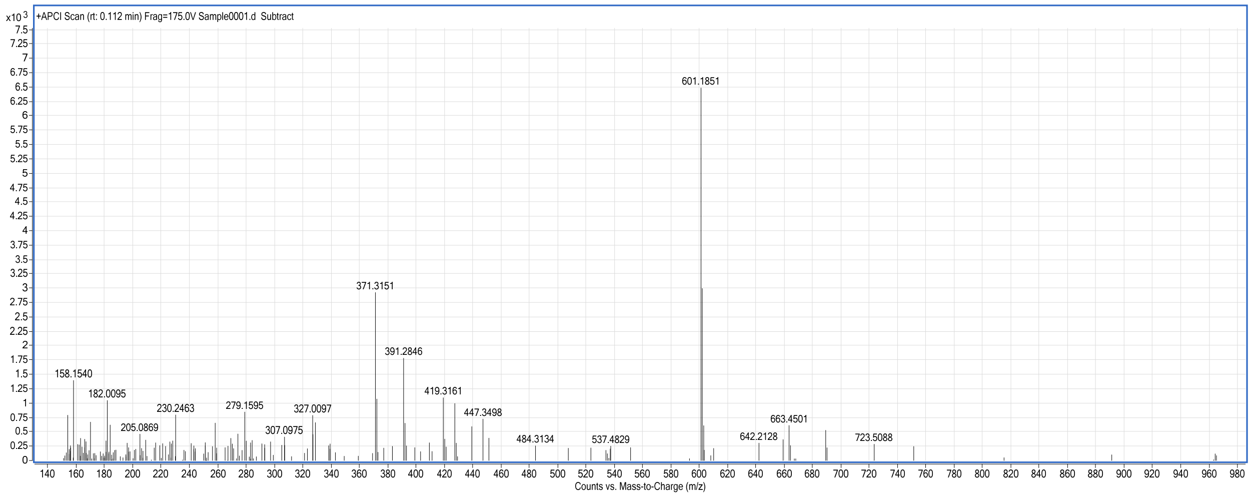

Figure S13. APCI for **1**.

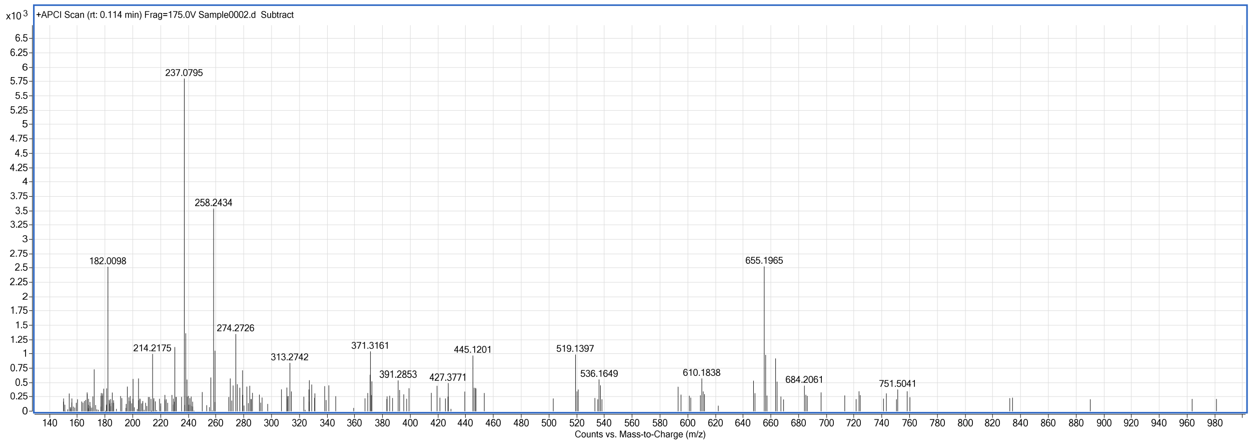

Figure S14. APCI for **2**.

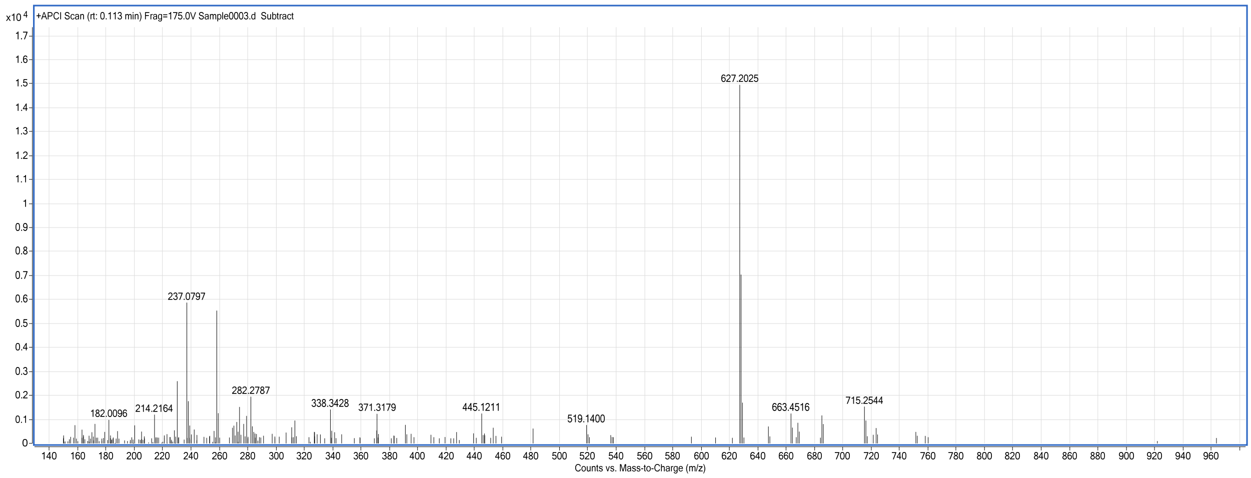

Figure S15. APCI for **3**.

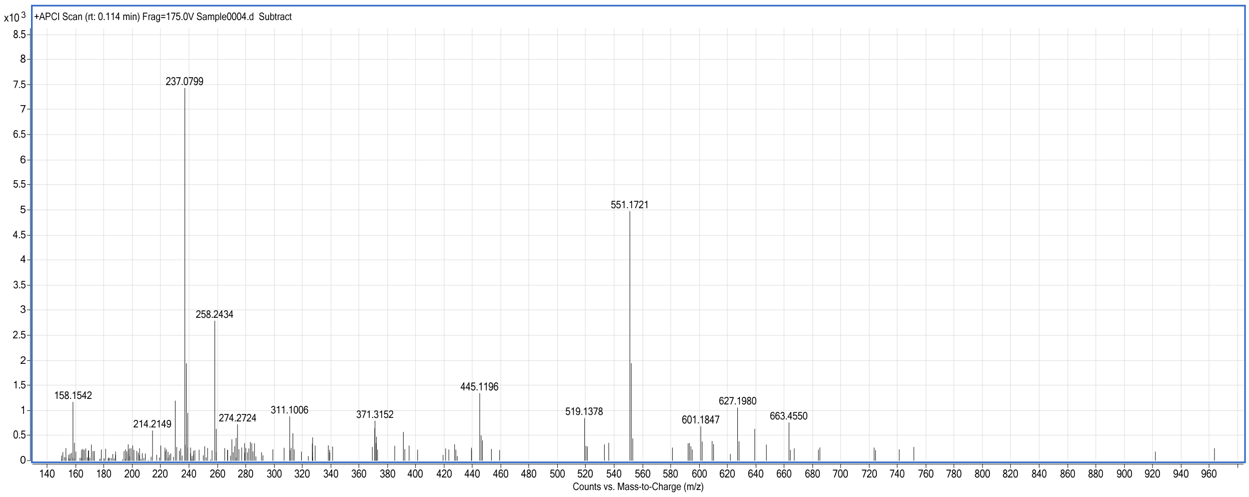

Figure S16. APCI for **4**.

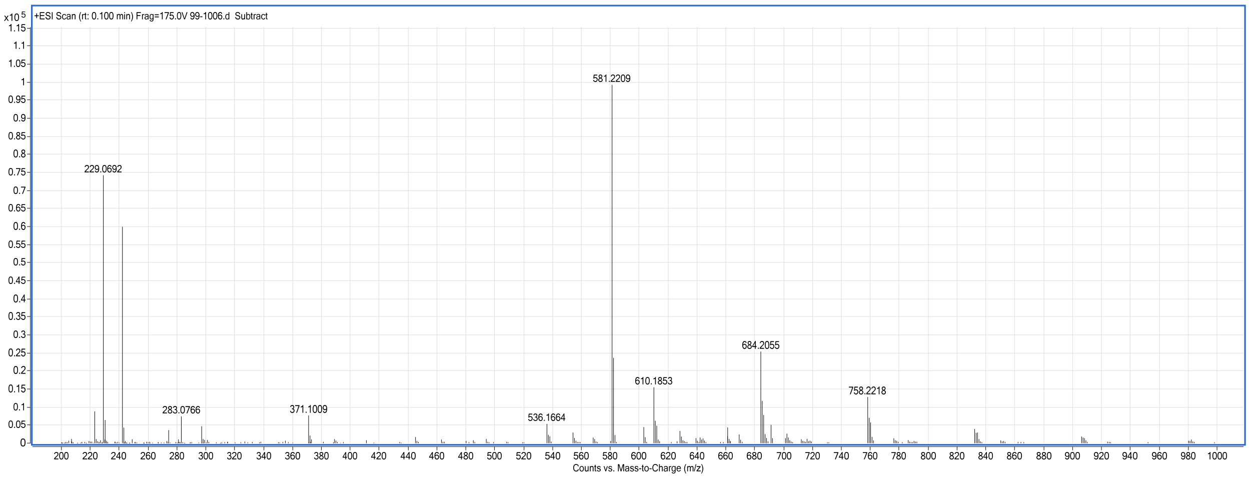

Figure S17. ESI for **5**.

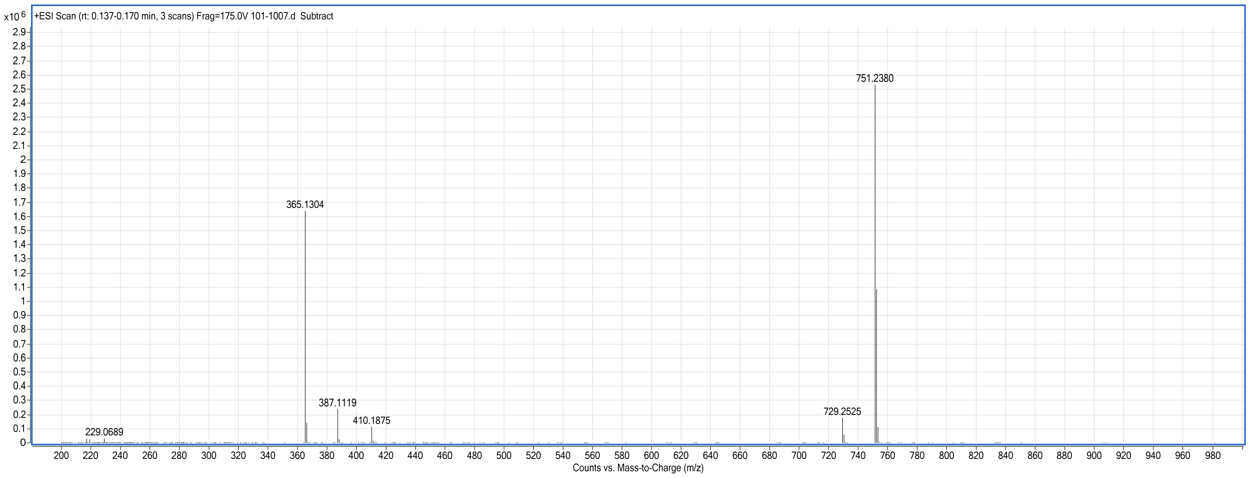

Figure S18. ESI for **6**.

S3. IR spectra

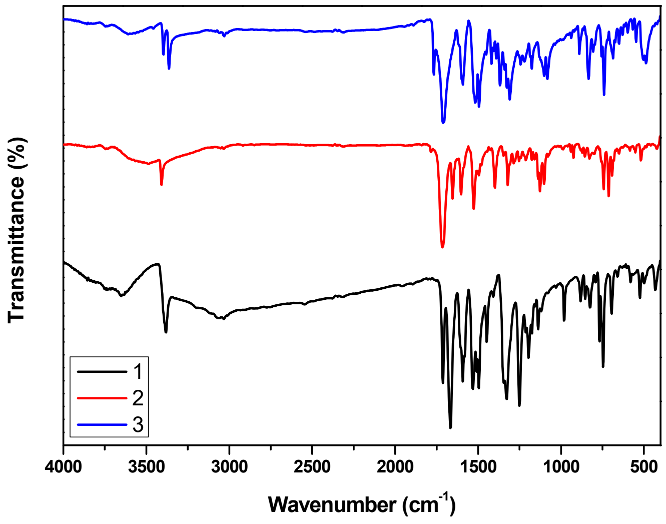

Figure S19. FT-IR spectra of **1**, **2**, and **3** (KBr pellets).

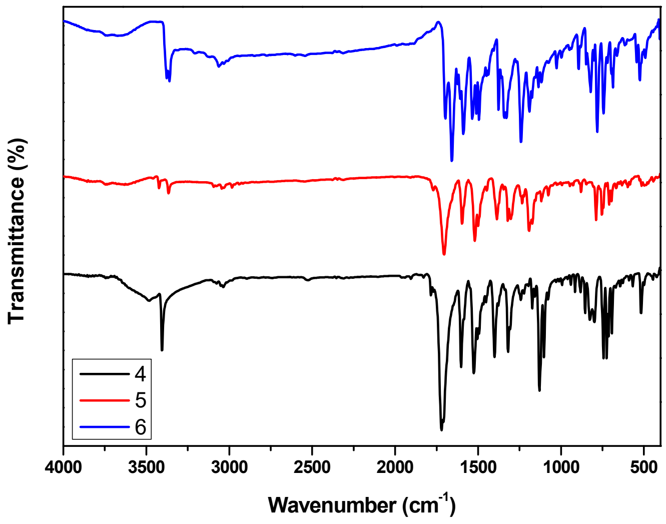

Figure S20. FT-IR spectra of **4**, **5**, and **6** (KBr pellets).

S4. TGA spectra

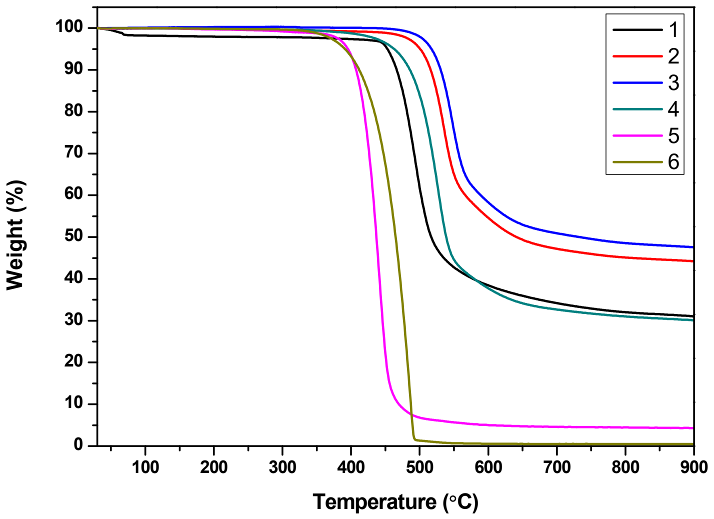

Figure S21. TGA curves for **1–6**.

S5. UV-vis spectra

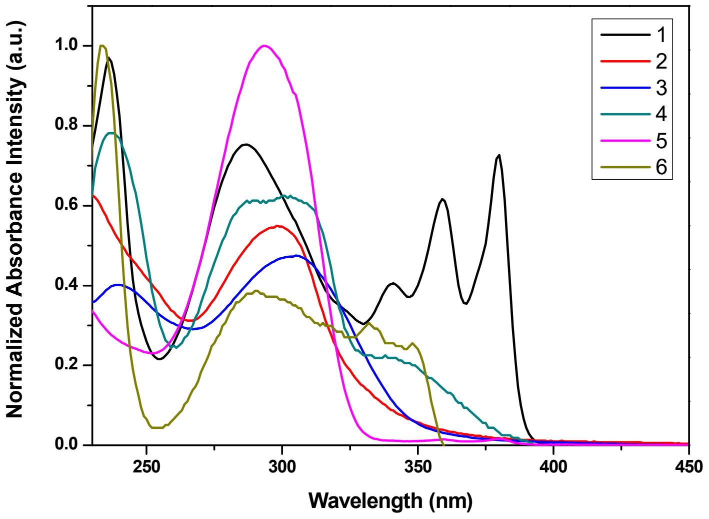

Figure S22. UV-vis spectra for **1–6** in DCM.

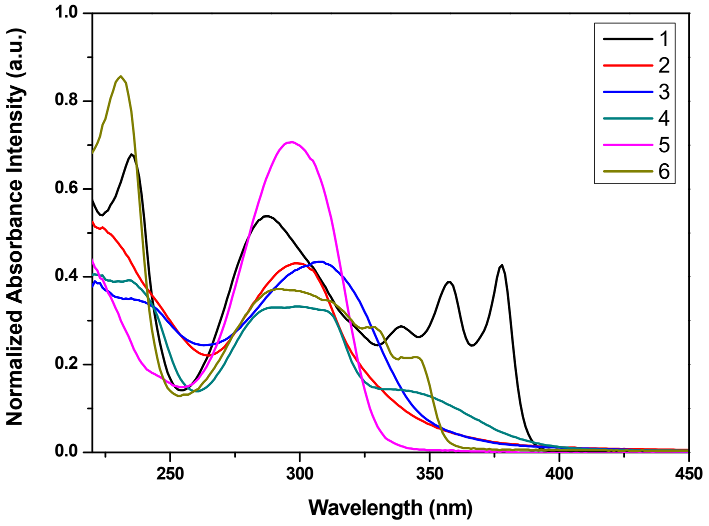

Figure S23. UV-vis spectra for **1–6** in THF.

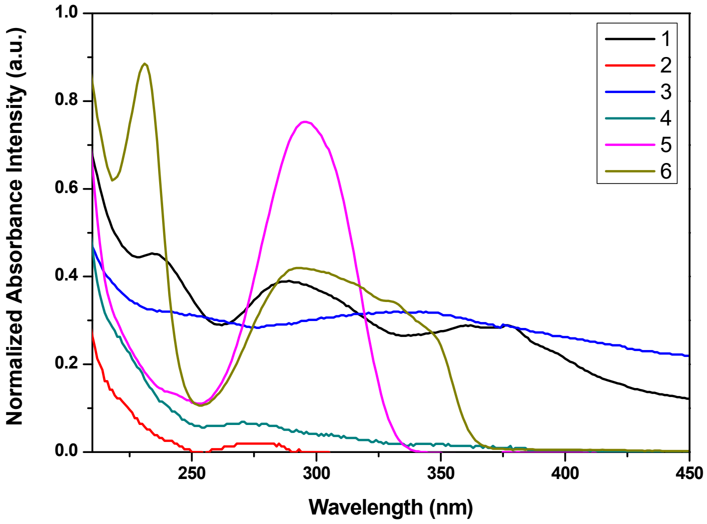

Figure S24. UV-vis spectra for **1–6** in EtOH.

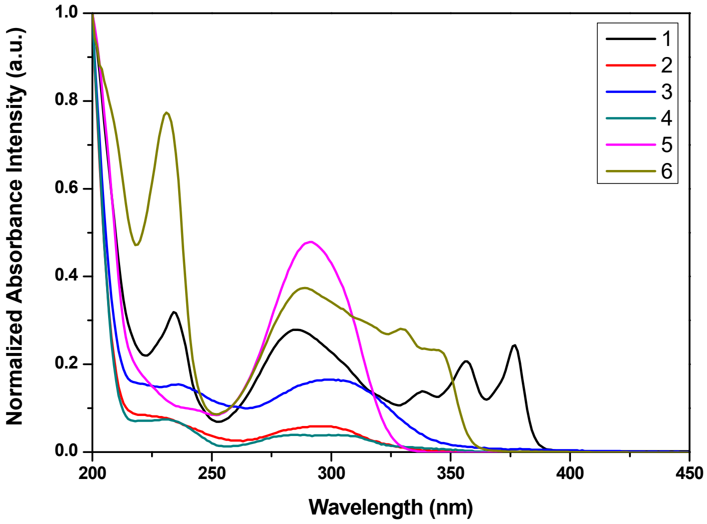

Figure S25. UV-vis spectra for **1–6** in CH_3CN.

S6. Fluorescence spectra

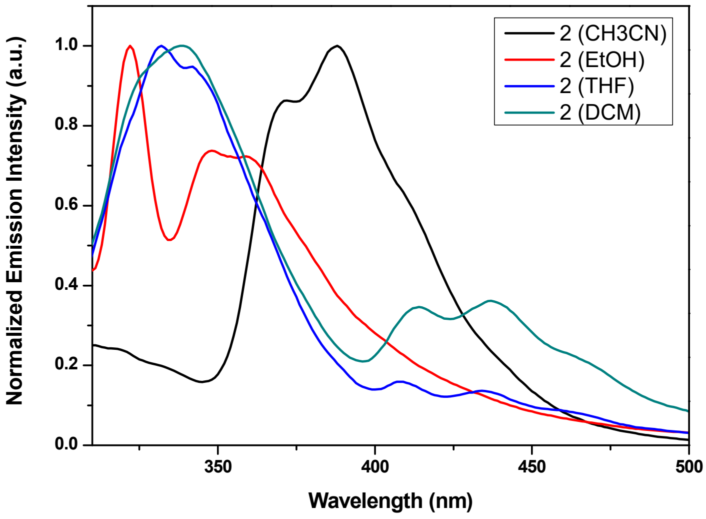

Figure S26. Normalized emission spectra of **2** were excited at 290 nm in CH_3CN (black), EtOH (red), THF (blue), and DCM (greenish blue), respectively at room temperature in air.

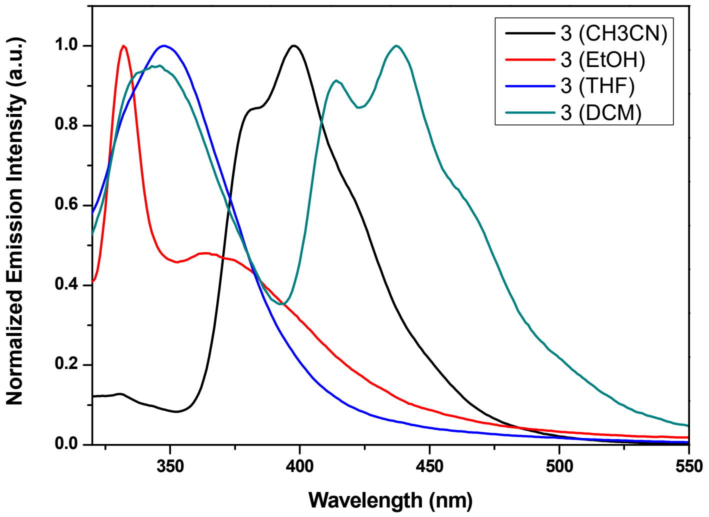

Figure S27. Normalized emission spectra of **3** were excited at 286 nm in CH_3CN (black), EtOH (red), THF (blue), and DCM (greenish blue), respectively at room temperature in air.

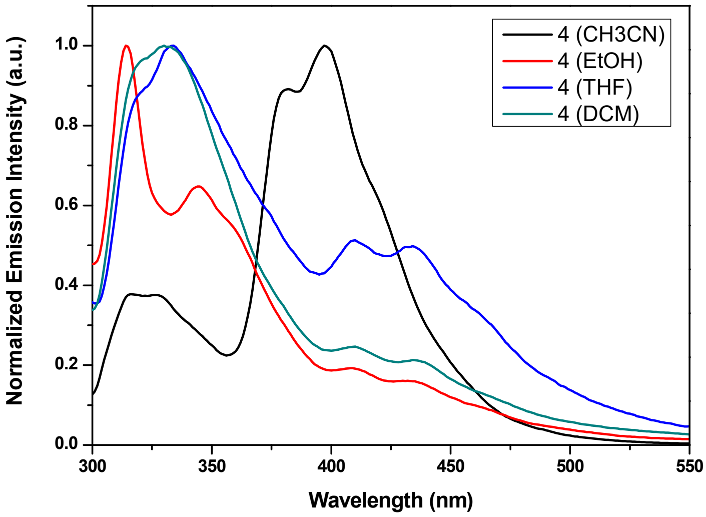

Figure S28. Normalized emission spectra of **4** were excited at 284 nm in CH_3CN (black), EtOH (red), THF (blue), and DCM (greenish blue), respectively at room temperature in air.

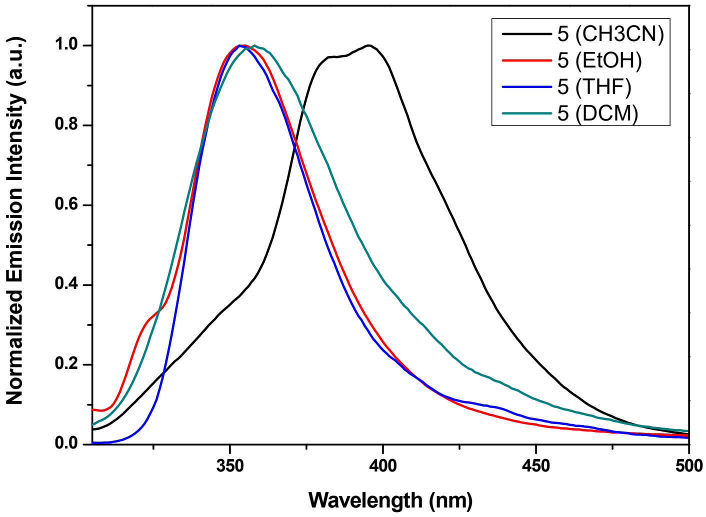

Figure S29. Normalized emission spectra of **5** were excited at 290 nm in CH_3CN (black), EtOH (red), THF (blue), and DCM (greenish blue), respectively at room temperature in air.

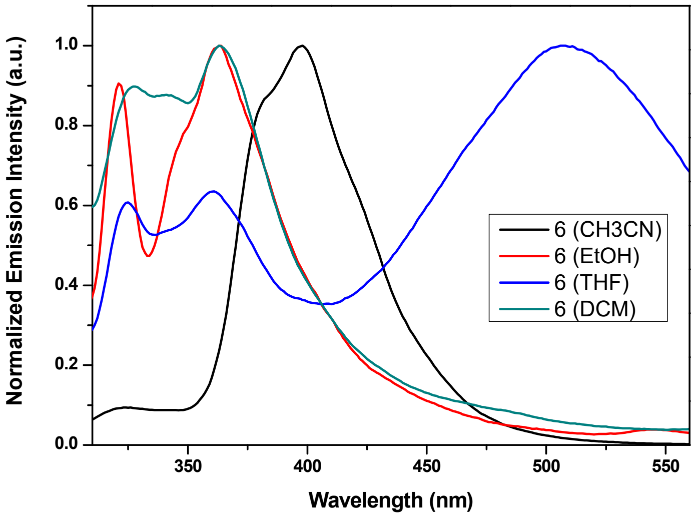

Figure S30. Normalized emission spectra of **6** were excited at 290 nm in CH_3CN (black), EtOH (red), THF (blue), and DCM (greenish blue), respectively at room temperature in air.

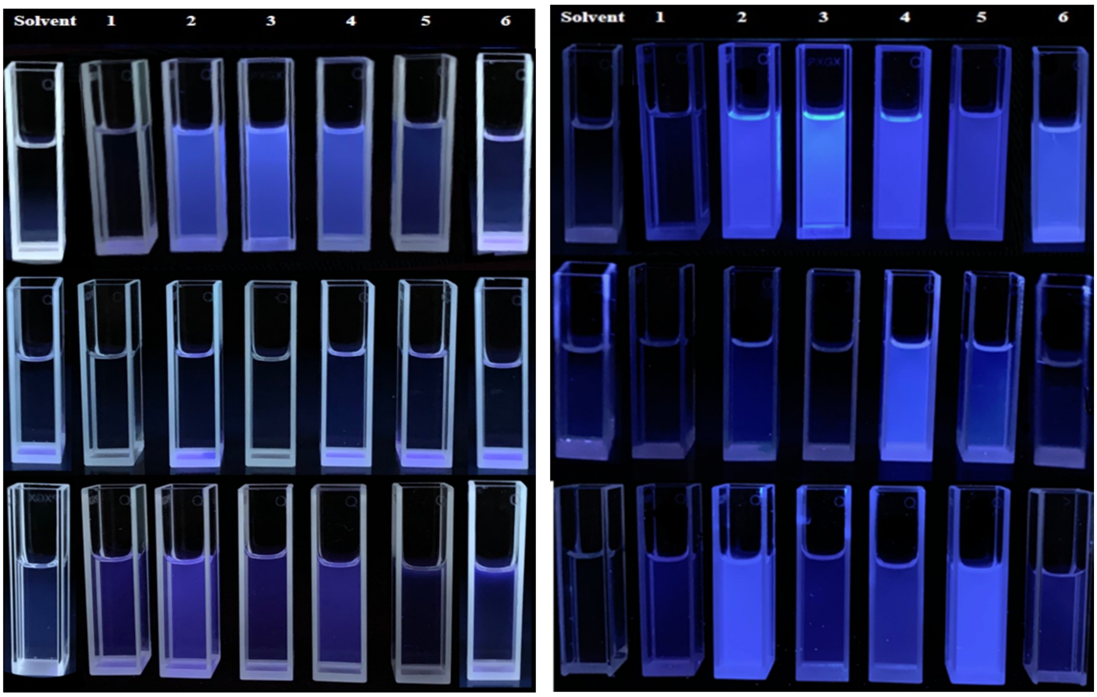

Figure S31. Emission photographs for **1–6** in DCM (top), EtOH (middle), and CH_3CN (bottom) excited at 254 nm (left) and 365 nm (right), respectively at room temperature in air.

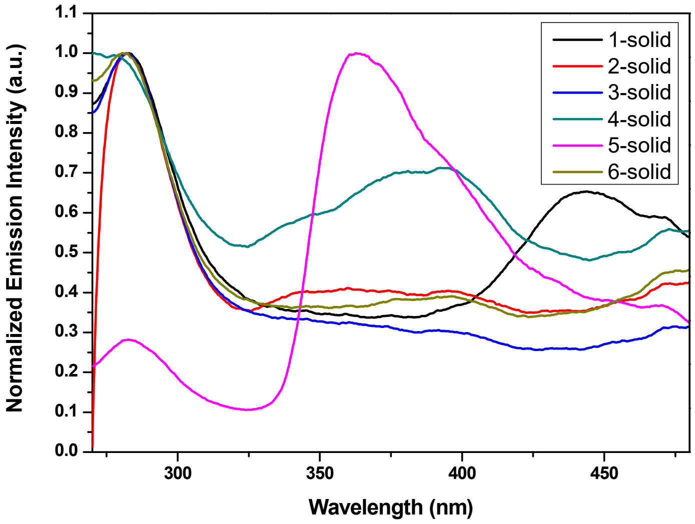

Figure S32. Solid state emission spectra excited at 250 nm for **1–6** at room temperature in air.

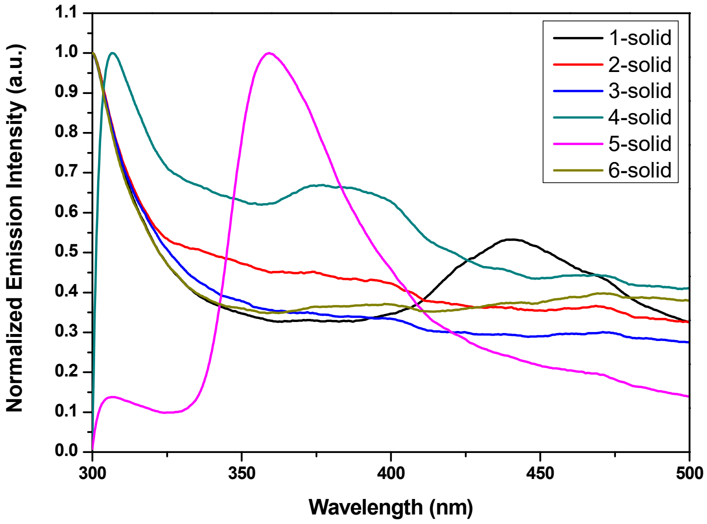

Figure S33. Solid state emission spectra excited at 280 nm for **1–6** at room temperature in air.

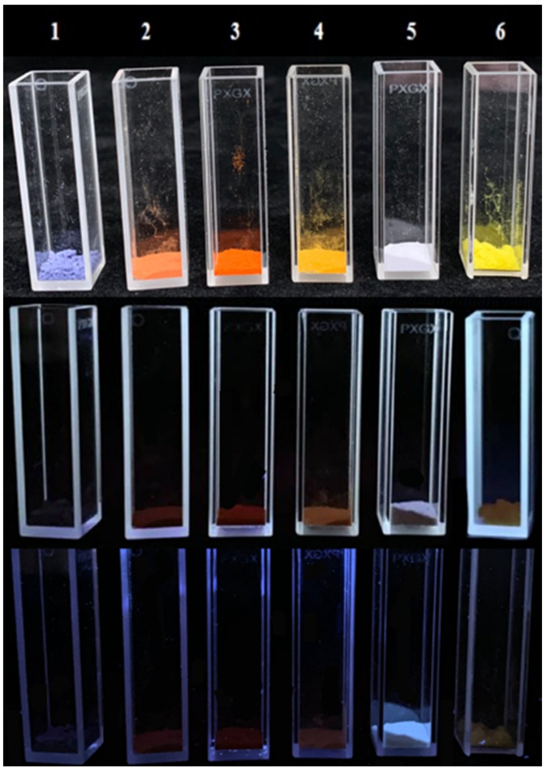

Figure S34. Emission photographs for **1–6** in solid state with regular light (top), excited at 365 nm (middle), and 254nm (bottom), respectively at room temperature in air.

S7. DPV and CV spectra

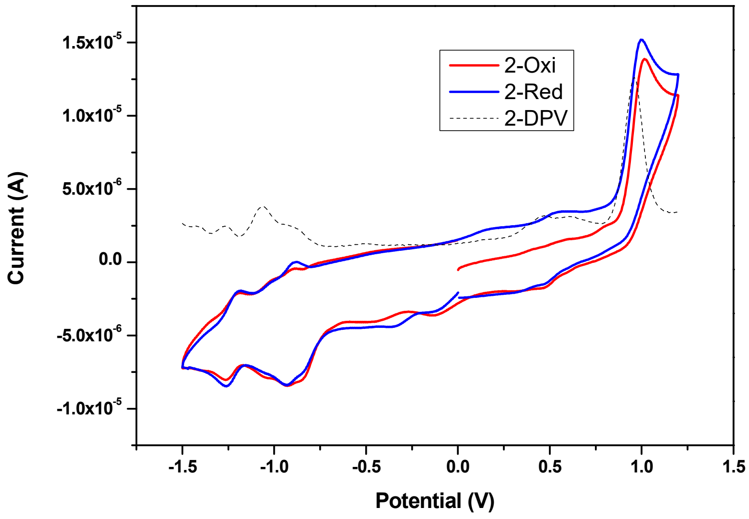

Figure S35. DPV in black dashed line and CV of **2** were measured in DMF with 0.1 M $TBAPF_6$. CV of 2-Oxi in red solid line indicated oxidation run first and 2-Red in blue solid line indicated reduction run first. Pt wire, Pt disk, and Ag/AgCl were used for measurements with a scan rate of 0.1 Vs^{-1}.

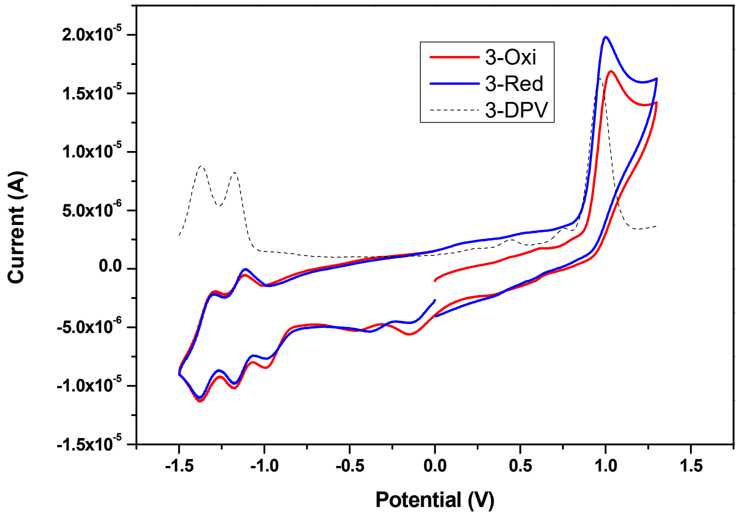

Figure S36. DPV in black dashed line and CV of **3** were measured in DMF with 0.1 M $TBAPF_6$. CV of 3-Oxi in red solid line indicated oxidation run first and 3-Red in blue solid line indicated reduction run first. Pt wire, Pt disk, and Ag/AgCl were used for measurements with a scan rate of 0.1 Vs^{-1}.

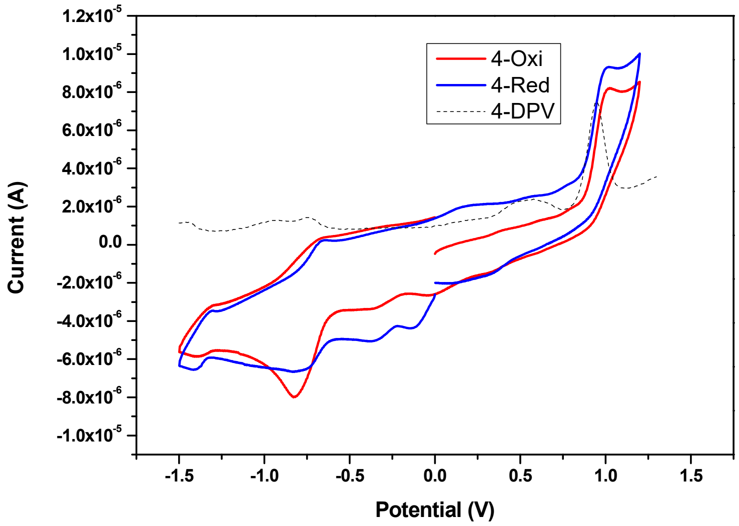

Figure S37. DPV in black dashed line and CV of **4** were measured in DMF with 0.1 M $TBAPF_6$. CV of 4-Oxi in red solid line indicated oxidation run first and 4-Red in blue solid line indicated reduction run first. Pt wire, Pt disk, and Ag/AgCl were used for measurements with a scan rate of 0.1 Vs^{-1}.

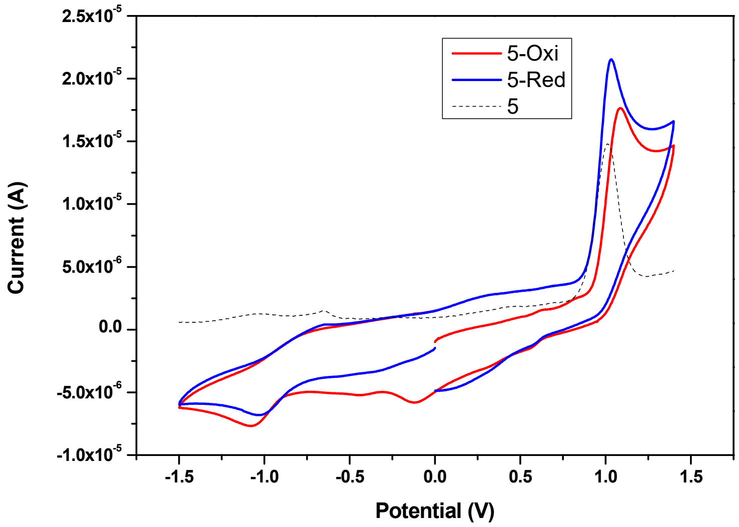

Figure S38. DPV in black dashed line and CV of **5** were measured in DMF with 0.1 M $TBAPF_6$. CV of 5-Oxi in red solid line indicated oxidation run first and 5-Red in blue solid line indicated reduction run first. Pt wire, Pt disk, and Ag/AgCl were used for measurements with a scan rate of 0.1 Vs^{-1}.

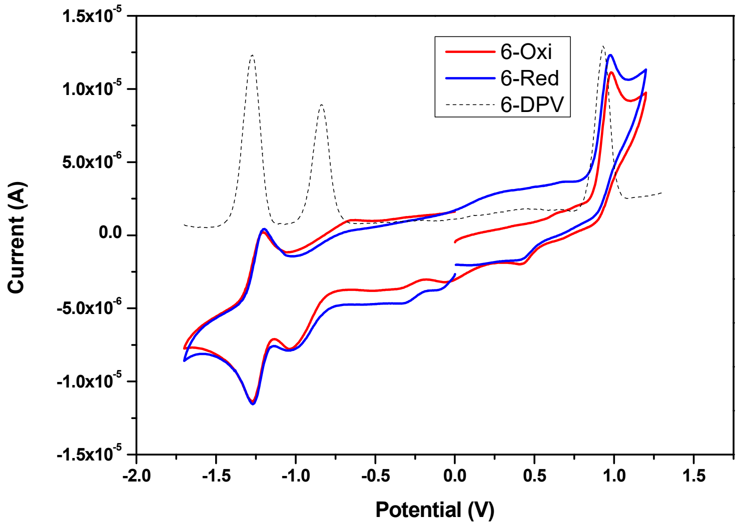

Figure S39. DPV in black dashed line and CV of **6** were measured in DMF with 0.1 M $TBAPF_6$. CV of 6-Oxi in red solid line indicated oxidation run first and 6-Red in blue solid line indicated reduction run first. Pt wire, Pt disk, and Ag/AgCl were used for measurements with a scan rate of 0.1 Vs^{-1}.

© 2019 by the authors. Submitted for possible open access publication under the terms and conditions of the Creative Commons Attribution (CC BY) license (http://creativecommons.org/licenses/by/4.0/).
